# Supplementary material for: Glucocorticoids paradoxically promote steroid resistance in B cell acute lymphoblastic leukemia through CXCR4/PLC signaling
Source: Nat Commun. 2024 May 29;15:4557. doi: 10.1038/s41467-024-48818-9 (PMC11136999; doi:10.1038/s41467-024-48818-9)
Supplement: Supplementary file 1 — Supplementary Information [file 41467_2024_48818_MOESM1_ESM.pdf]

Glucocorticoids paradoxically promote steroid resistance in B cell acute lymphoblastic leukemia through CXCR4/PLC signaling

Souleymane Abdoul-Azize[1]*, Rihab Hami[2], Gaetan Riou[1], Céline Derambure[3], Camille Charbonnier[3], Jean-Pierre Vannier[1], Monica L Guzman[4], Pascale Schneider[1,5], and Olivier Boyer[1,6]

[1]Univ Rouen Normandie, Inserm, UMR 1234, F-76000 Rouen, France
[2]Univ Brest, Inserm, UMR 1101, F-29200 Brest, France
[3]Univ Rouen Normandie, Inserm, UMR 1245, Rouen, France
[4]Division of Hematology and Oncology, Department of Medicine, Weill Cornell Medicine, New York, NY, USA
[5]Rouen University Hospital, Department of Pediatric immuno-hemato-oncology, F-76000 Rouen, France.
[6]Rouen University Hospital, Department of Immunology and Biotherapy, F-76000 Rouen, France

*Corresponding author: souleymane.abdoulazize@inserm.fr

Supplementary information

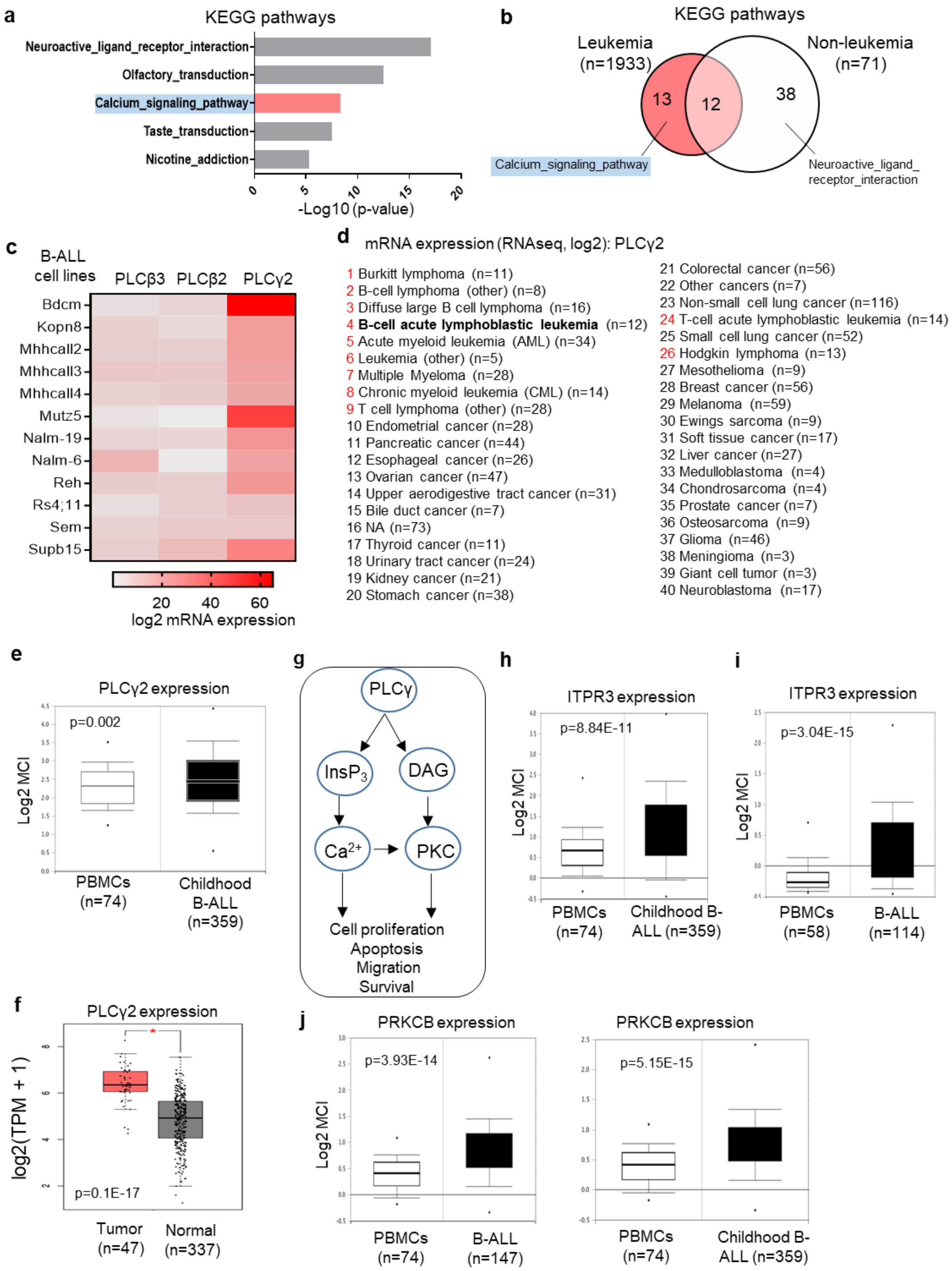

Supplementary Figure 1. PLCγ2 is highly expressed in B-ALL cells.

(**a**) Overrepresented pathways from the Kyoto Encyclopedia of Genes and Genomes (KEGG) whose genes are differentially expressed between healthy (n=71 patients) and leukemic (n=1933 patients) groups in the MILE dataset from R2 database. Top-5 pathways are shown and ranked by p-value from statistical analysis by two-tailed, *t*-test. (**b**) Venn diagram shows summary of KEGG pathway analysis between non-leukemia and leukemic samples from panel (**a**). An enriched pathway is indicated for each subset. Statistical analysis

by two-tailed, *t*-test, p-value < 0.05 for all pathways. (**c**) Expression of PLCβ2, PLCβ3, and PLCγ2 genes from different B-ALL cell lines. Heat map shows absolute mRNA expression. Data were extracted from the cancer cell line encyclopedia (CCLE). (**d**) Human cancer cell lines from the CCLE database, ranked by PLCγ2 mRNA level (high to low). Related to Figure 1**b**. Numbering is the same as in Figure 1**b**. Hematological malignancies are depicted in red. 'n' represents the number of cell lines analyzed in each cancer type. (**e**) PLCγ2 mRNA level (median-centered intensity, MCI) graphed as mean with SD between PBMCs and B-ALL samples in Haferlach Leukemia data set. Data were obtained from the Oncomine portal. Statistical analysis by two-sided *t*-test. (**f**) PLCγ2 mRNA level graphed as mean with SD between tumor and normal samples in diffuse large B cell lymphoma from the Gene Expression Profiling Interactive Analysis (GEPIA) database. Statistical significance was analyzed by one-way ANOVA. (**g**) Schematic diagram of the PLC signaling pathway. Inositol Trisphosphate (IP_3), Diacylglycerol (DAG), Protein kinase c (PKC). (**h**,**i**) ITPR3 mRNA level (median-centered intensity, MCI) graphed as mean with SD between PBMCs and B-ALL samples in Haferlach Leukemia (**h**) and Haferlach Leukemia 2 (**i**) data sets, respectively. Data were obtained from the Oncomine portal. Statistical analysis by two-sided *t*-test. (**j**) PRKCB mRNA level (median-centered intensity, MCI) graphed as mean with SD between PBMCs and B-ALL samples in Haferlach Leukemia data set. Data were obtained from the Oncomine portal. Statistical analysis by two-sided *t*-test. Source data are provided as a Source Data file.

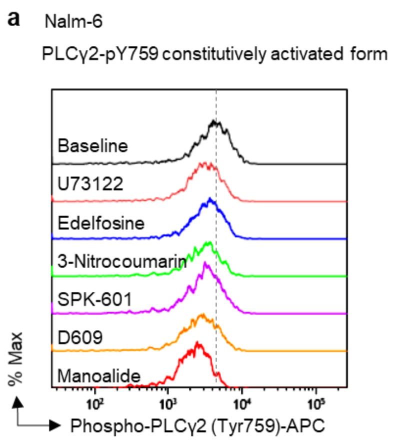

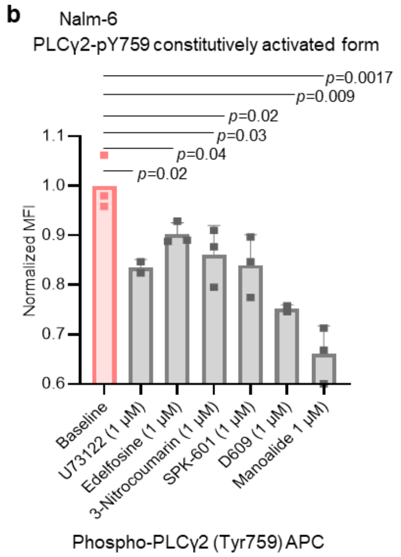

d Cch Ca^{2+} release

Two-Way Anova with Sidak's multiple comparisons test	Mean Diff	Summary	p-value
**Nalm-6 cells**			
Ctr vs. U73122	0.1337	***	0.0001
Ctr vs. Edelfosine	0.1574	****	<0.0001
Ctr vs. 3-Nitrocoumarin	0.1287	***	0.0002
Ctr vs. SPK-601	0.1102	**	0.0013
Ctr vs. D609	0.1403	****	<0.0001
Ctr vs. Manoalide	0.1461	****	<0.0001
**RS4;11 cells**			
Ctr vs. U73122	0.07928	*	0.0296
Ctr vs. Edelfosine	0.0843	*	0.0186
Ctr vs. 3-Nitrocoumarin	0.1114	**	0.0013
Ctr vs. SPK-601	0.08945	*	0.0114
Ctr vs. D609	0.08333	*	0.0203
Ctr vs. Manoalide	0.07389	*	0.0484

e ATP Ca^{2+} release

Two-Way Anova with Sidak's multiple comparisons test			
**ATP**	**Mean Diff**	**Summary**	**p-value**
**Nalm-6 cells**			
Ctr vs. U73122	0.07146	*	0.0109
Ctr vs. Edelfosine	0.09731	***	0.0004
Ctr vs. 3-Nitrocoumarin	0.1183	****	<0.0001
Ctr vs. SPK-601	0.0865	**	0.0017
Ctr vs. D609	0.1002	***	0.0003
Ctr vs. Manoalide	0.1203	****	<0.0001
**RS4;11 cells**			
Ctr vs. U73122	0.08909	**	0.0012
Ctr vs. Edelfosine	0.1226	****	<0.0001
Ctr vs. 3-Nitrocoumarin	0.1009	***	0.0003
Ctr vs. SPK-601	0.1025	***	0.0002
Ctr vs. D609	0.1027	***	0.0002
Ctr vs. Manoalide	0.07277	**	0.0093

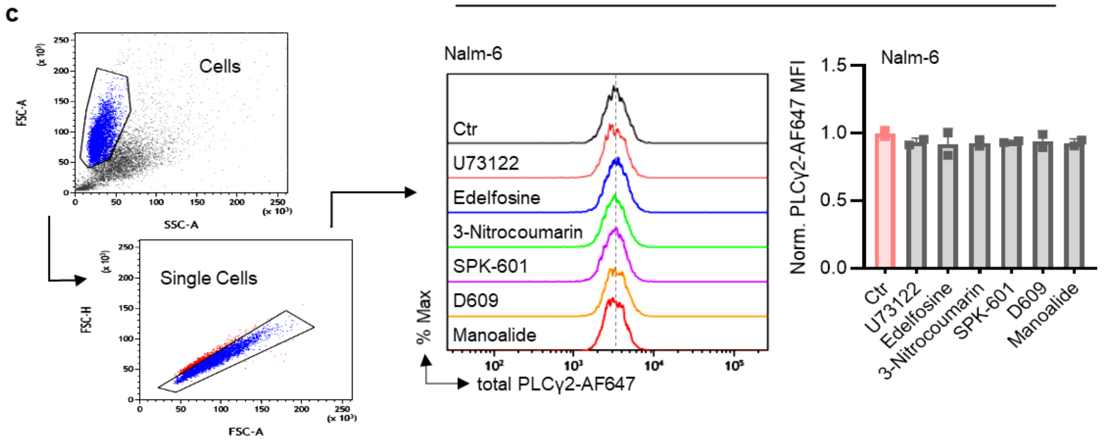

Supplementary Figure 2. PLCγ2 is constitutively activated in B-ALL cells.

(**a**) Representative flow cytometry plots of the active form of PLCγ2 (phospho-PLCγ2 (Tyr759)) in Nalm-6 cells treated with PLC inhibitors or Ctr (Baseline) for 24 hours. (**b**) Quantification from panel (**a**) of the active form of PLCγ2 (phospho-PLCγ2 (Tyr759)) normalized to Ctr (Baseline). Statistical significance was analyzed by two-tailed, unpaired Student's *t* test. Data shown are the mean ± SEM of n = 3 independent experiments. (**c**) Representative gating strategy (left), flow cytometry plots (middle) and quantification (right) of the total form of PLCγ2 in Nalm-6 cells treated with PLC inhibitors or Ctr for 24 hours and normalized to Ctr. Data are mean ± SEM (n=2 independent experiments). (**d**,**e**) P-values based on two-way ANOVA with Sidak's multiple comparisons test related to Figure 1**j** and 1**k**, respectively. Source data are provided as a Source Data file.

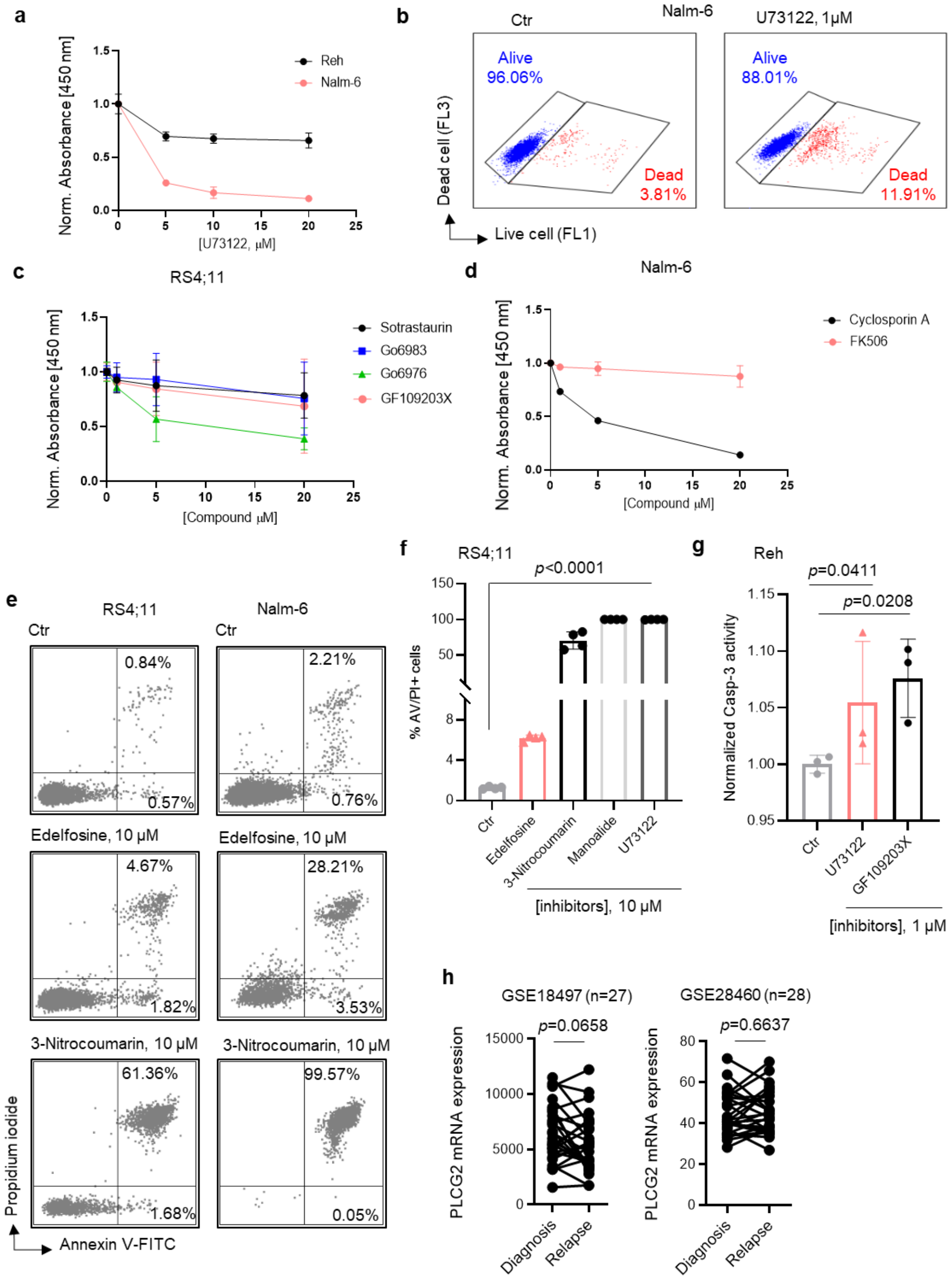

Supplementary Figure 3. PLC pathway inhibition triggers cytotoxicity in B-ALL cell lines

(**a**) Viability normalized to the solvent control of Reh and Nalm-6 cells exposed for 48 hours with increasing concentrations of the PLC inhibitor, U73122. Data are presented as means ± SEM (n=4 independent experiments). (**b**) Nalm-6 cells treated with 1 µM U73122 for 48 hours of treatment. Alive and dead cells were determined by flow cytometry after LIVE/DEAD staining. (**c**) Viability normalized to the solvent control of RS4;11 cells exposed for 48 hours with increasing concentrations of four PKC inhibitors (Sotrastaurin, Go6983, Go6976 and GF109203X). Data are presented as means ± SEM of n=5 independent

experiments. (**d**) Viability normalized to the solvent control of Nalm-6 cells exposed for 48 hours with increasing concentrations of two calcineurin inhibitors (Cyclosporin A and FK506). Data are presented as means ± SEM (n=4 independent experiments). (**e**) Nalm-6 and RS4;11 cells treated with four PLC inhibitors at 10 μM. Cell mortality was determined by flow cytometry and annexin V/PI staining after 48 hours of treatment followed by FACS analysis. Data are representative of 3 independent experiments. (**f**) Annexin V/PI positive cells were measured by flow cytometry after 24 hours of Ctr, U73122, Edelfosine, 3-Nitrocoumarin, and Manoalide treatment. Data are presented as means ± SEM (n=4 independent experiments). Statistical significance was analyzed by two-tailed, unpaired Student's t test. (**g**) Caspase-3 activity was determined using Ac-DEVD-AFC as substrate after 24 hours of Ctr, U73122 and GF109203X treatment. Data are presented as means ± SEM (n=3 independent experiments). Statistical significance was analyzed by two-tailed, unpaired Student's t test. (**h**) PLCγ2 mRNA level between diagnosis and relapse B-ALL group in publicly available transcriptomic datasets (n=27 patients for GSE18497, n=28 patients for GSE28460). Statistical significance was analyzed by two-tailed, paired t test. Source data are provided as a Source Data file.

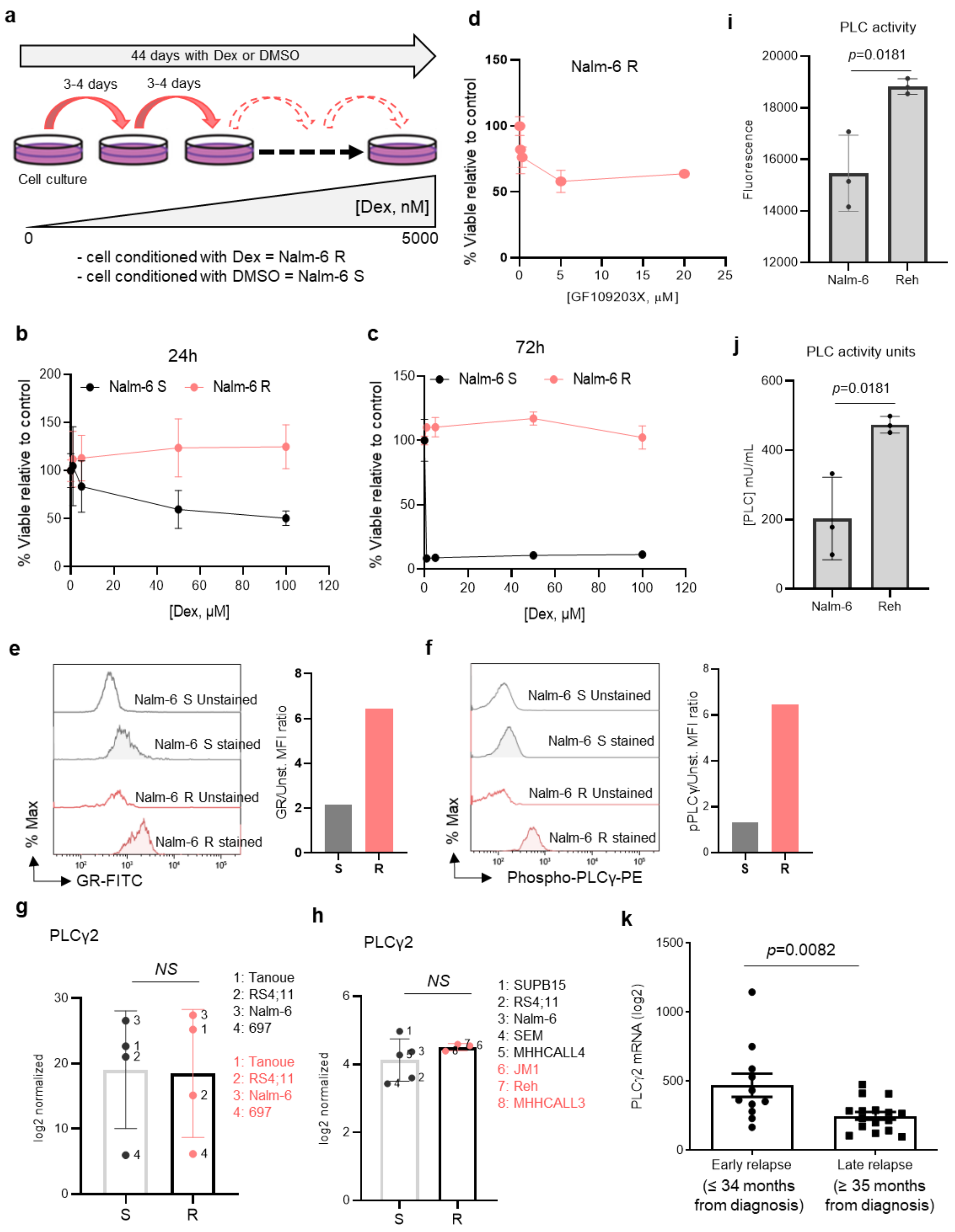

Supplementary Figure 4. PLC is more active in resistant B-ALL cells

(**a**) Cell culture method for generating Dex-resistant B-ALL cells. Nalm-6 cells were treated with increasing concentrations (from 1 to 5000 nM) of Dex for 44 days. (**b**,**c**) Viability relative to Ctr of Dex-resistant Nalm-6 cells (named as Nalm-6 R) and Dex-sensitive Nalm-6 cells (named as Nalm-6 S), exposed for 24 (**b**) or 72 (**c**) hours with increasing concentrations of Dex. Data are mean ± SEM from (**b**: n = 4 from 0 µM to 5 µM and n=2 for 50 µM and 100 µM in Nalm-6 S and n=4 for Nalm-6 R, **c**: n = 4 in Nalm-6 R except for 50 µM with n=2

and n=4 for Nalm-6 S; n-values correspond to independent experiments). (**d**) Viability relative to control of Nalm-6 R exposed for 48 hours with increasing concentrations of PKC inhibitor, GF109203X. Data are mean ± SEM from (n = 4 from 0 μM to 0.3 μM and n=2 for 5 μM and 20 μM; n-values correspond to independent experiments). (**e**) Flow cytometry plots of glucocorticoid receptor (GR) of Nalm-6 S and Nalm-6 R cells. (**f**) Flow cytometry plots of the constitutively activated form of PLCγ2 (phospho-PLCγ2) of Nalm-6 S and Nalm-6 R cells. (**g**) PLCγ2 mRNA level between sensitive B-ALL and their parental resistant cell lines in publicly available transcriptomic dataset. Statistical significance was analyzed by two-tailed, unpaired Student's t test. Data shown are the mean ± SD of n = 4 cell lines. (**h**) PLCγ2 mRNA level between sensitive B-ALL cell lines and resistant B-ALL cell lines in publicly available transcriptomic dataset. Statistical significance was analyzed by two-tailed, unpaired Student's t test. Data shown are the mean ± SD of (n = 5 sensitive, S, cell lines; n= 3 resistant, R, cell lines). (**i**,**j**) Quantification of PLC activity (**i**) and intracellular concentration of PLC (mU/mL) (**j**) in Nalm-6 and Reh cells. Statistical significance was analyzed by two-tailed, unpaired Student's t test. Data are mean ± SEM of n=3 independent experiments measured by ELISA. (**k**) PLCγ2 mRNA level between early (n=11 patients) and late (n=16 patients) relapse B-ALL group in publicly available transcriptomic dataset. Data are mean ± SD. Statistical significance was analyzed by two-tailed, unpaired Student's t test. S: sensitive, R: resistant. Source data are provided as a Source Data file.

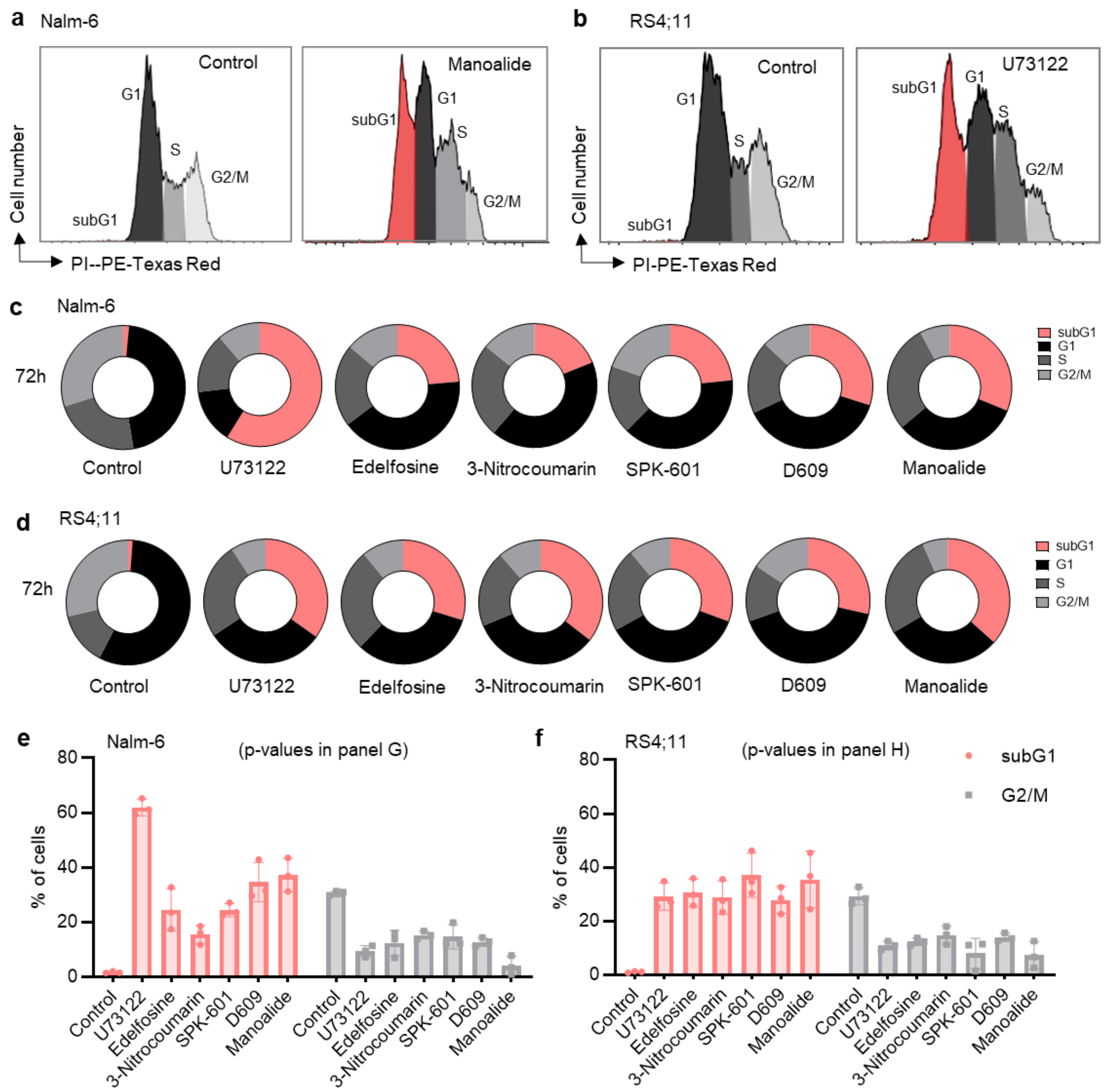

g

Two-tailed unpaired Student's t test		
	subG1 p-value	G2/M p-value
**Nalm-6 cells**		
Ctr vs. U73122	<0.0001	<0.0001
Ctr vs. Edelfosine	0.0068	0.0025
Ctr vs. 3-Nitrocoumarin	0.0022	<0.0001
Ctr vs. SPK-601	<0.0001	0.0037
Ctr vs. D609	0.0014	<0.0001
Ctr vs. Manoalide	0.0006	0.0002

h

Two-tailed unpaired Student's t test		
	subG1 p-value	G2/M p-value
**RS4;11 cells**		
Ctr vs. U73122	0.0007	0.0009
Ctr vs. Edelfosine	0.0006	0.0011
Ctr vs. 3-Nitrocoumarin	0.0015	0.0056
Ctr vs. SPK-601	0.0016	0.0047
Ctr vs. D609	0.0008	0.0017
Ctr vs. Manoalide	0.0048	0.0031

Supplementary Figure 5. PLC inhibitors provoke cell cycle arrest in human B-ALL cell lines.

(**a-d**) Cell cycle analysis of Nalm-6 and RS4;11 cells before and after stimulation with PLC inhibitors at 5 µM for 72 hours. Representative flow cytometry plots (**a**,**b**) and proportion (**c**,**d**) of different cell cycle phases. (**e**,**f**) Quantification of subG1 and G2/M cell cycle phases from panel **c** and **d**, respectively. Data are represented as mean ± SEM of n=3 independent experiments. Statistical significance was analyzed by two-tailed, paired t test. (**g**,**h**) P-values of subG1 and G2/M cell cycle phases were determined by two-tailed, unpaired Student's t test, from panel **e** and **f**, respectively. Data are mean ± SEM (n=3 independent experiments). Source data are provided as a Source Data file.

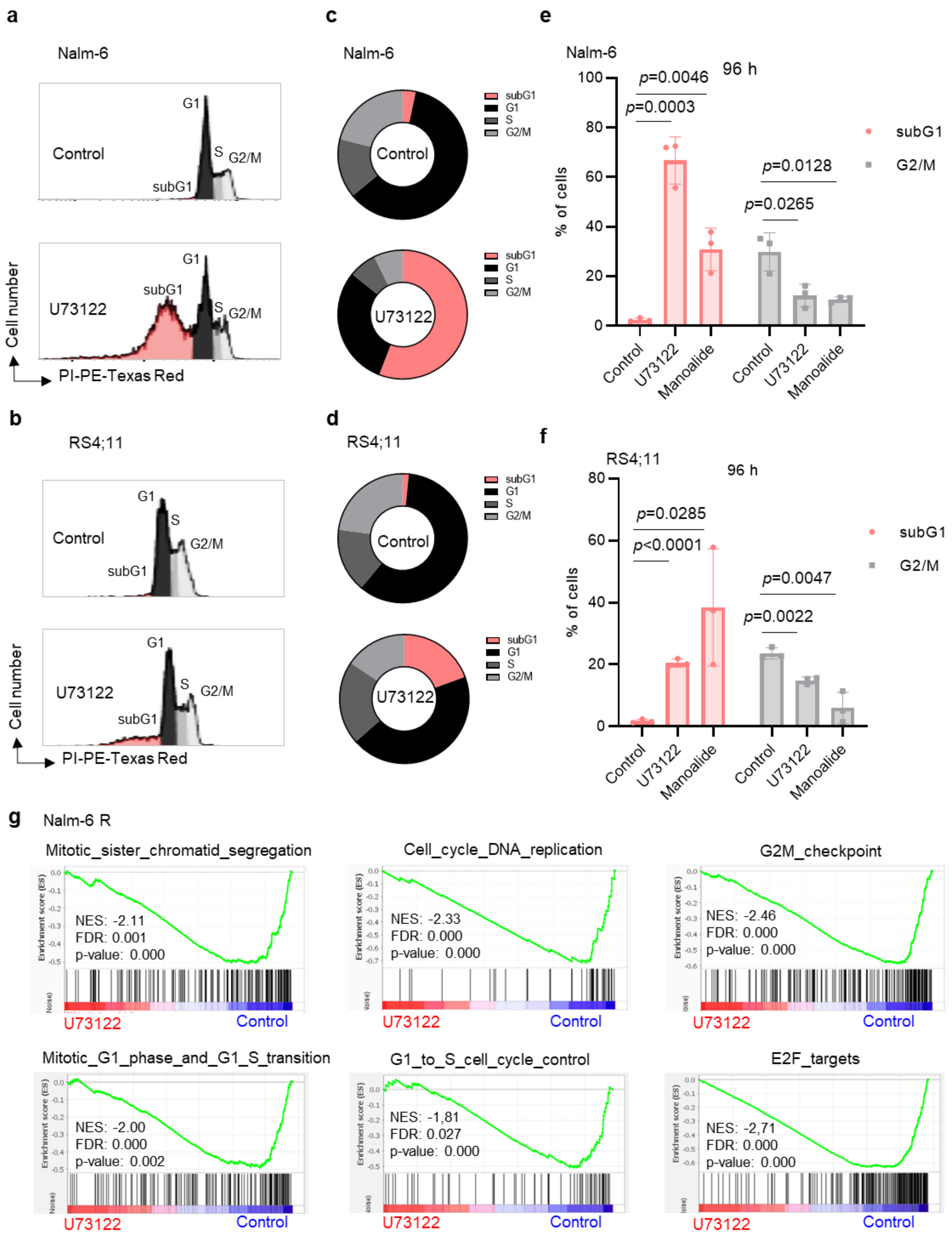

Supplementary Figure 6. PLC inhibiton impaired cell cycle checkpoints in human B-ALL cell lines.

(**a-d**) Cell cycle analysis of Nalm-6 and RS4;11 cells before and after stimulation with PLC inhibitors at 5 µM for 96 hours. Representative flow cytometry plots (**a**,**b**) and proportion (**c**,**d**) of different cell cycle phases. **(e,f)** Quantification of subG1 and G2/M cell cycle phases from panel **c** and **d**, respectively. Statistical significance was analyzed by two-tailed, unpaired Student's t test. Data are mean ± SEM (n=3 independent experiments). (**g**) GSEA plots of RNA-seq (n=2 biological replicates) showing Mitotic sister chromatid segregation, Cell_cycle DNA replication, G2M checkpoint, Mitotic G1 phase and G1 S transition, G1 to S cell cycle control and E2F target signatures comparing Ctr versus U73122 in Dex-resistant Nalm-6 cells after 16 hours of treatment. Source data are provided as a Source Data file.

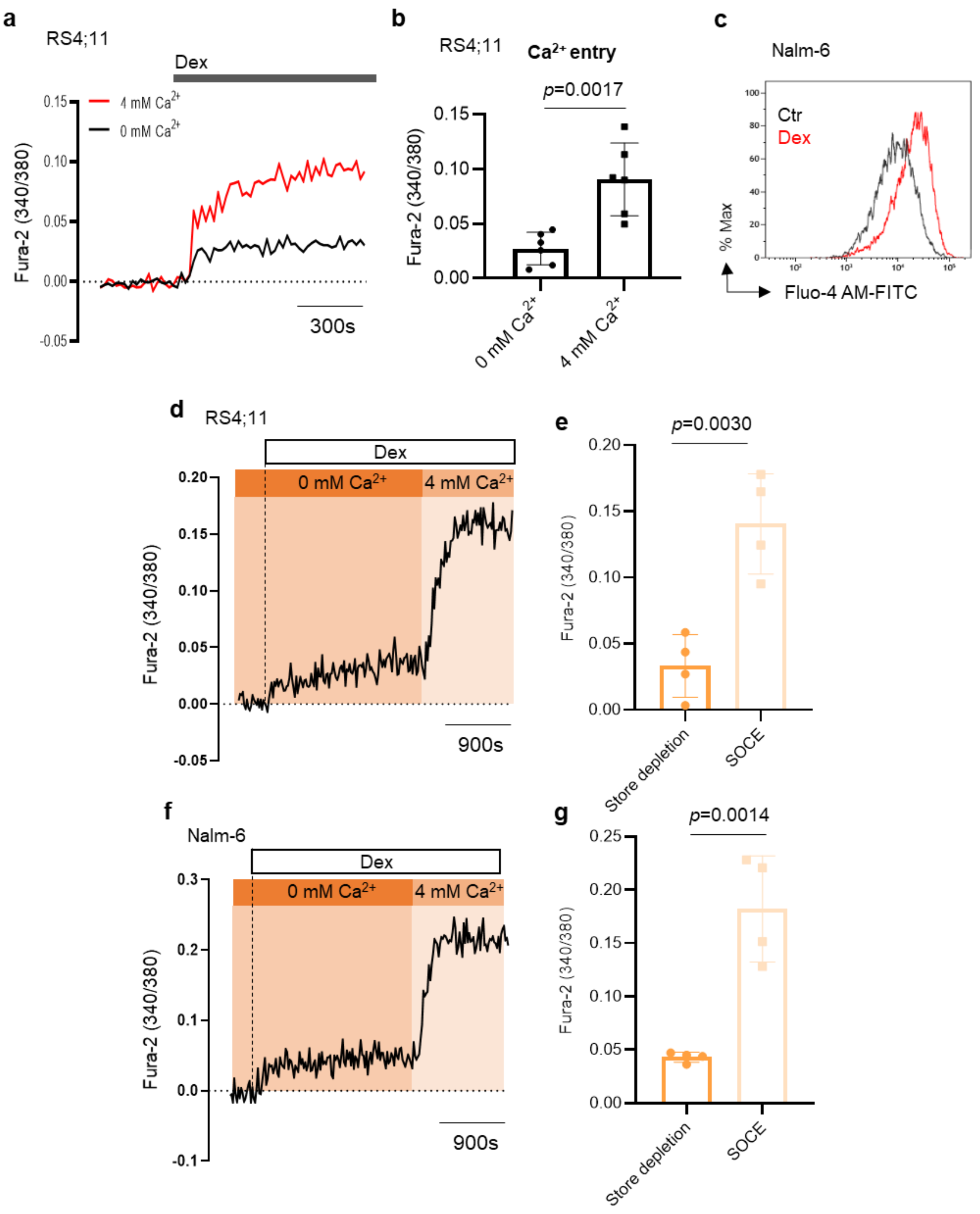

Supplementary Figure 7. Dex stimulates intracellular Ca^{2+} release and SOCE in B-ALL cells.

(**a**) Cytosolic Ca^{2+} measurements in RS4;11 cells stimulated with 125 nM Dex in nominally Ca^{2+}-free buffer (0 mM Ca^{2+}) and Ca^{2+}-containing buffer (4 mM Ca^{2+}). (**b**) Quantification of maximal Ca^{2+} entry from panel **a.** Data are represented as mean ± SEM of n=6 independent experiments. (**c**) Flow cytometry plots of Nalm-6 cells stimulated with 125 nM Dex or vehicle Ctr using Fluo-4 AM probe. (**d-f**) Cytosolic Ca^{2+} measurements in RS4;11 (**c**) and Nalm-6 (**e**). Fura-2-loaded cells were stimulated by Dex (125 nM) in Ca^{2+}-free buffer followed by re-addition of extracellular Ca^{2+} (4 mM Ca^{2+} containing buffer). (**e-g**) Quantification of Ca^{2+} traces in RS4;11 (**d**) and Nalm-6 (**f**) cells under the conditions indicated, store depletion (0 mM Ca^{2+}) and SOCE (4mM Ca^{2+}). Data are represented as mean ± SEM of n=4 independent experiments. Statistical significance was analyzed by two-tailed, unpaired Student's t test. Source data are provided as a Source Data file.

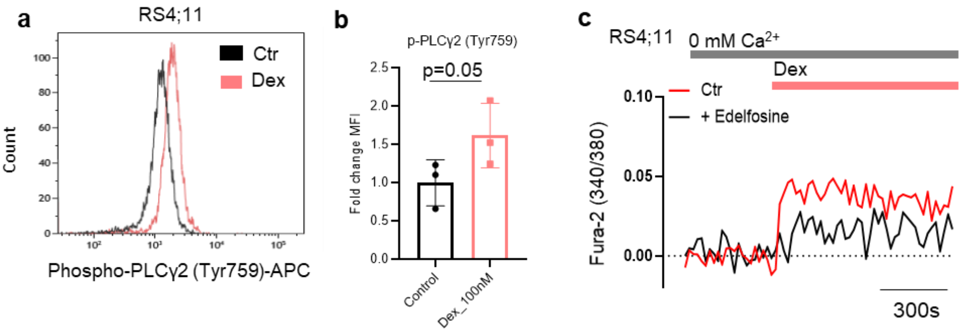

d

Dex Ca^{2+} entry

Two-Way Anova with Sidak's multiple comparisons test	Mean Diff	Summary	p-value
Nalm-6 cells			
Ctr vs. U73122	0.0413	*	0.0491
Ctr vs. Edelfosine	0.05911	**	0.0024
Ctr vs. 3-Nitrocoumarin	0.06204	**	0.0014
Ctr vs. SPK-601	0.06842	***	0.0005
Ctr vs. D609	0.07306	***	0.0002
Ctr vs. Manoalide	0.07168	***	0.0003
RS4;11 cells			
Ctr vs. U73122	0.08912	****	<0.0001
Ctr vs. Edelfosine	0.1215	****	<0.0001
Ctr vs. 3-Nitrocoumarin	0.08596	****	<0.0001
Ctr vs. SPK-601	0.1218	****	<0.0001
Ctr vs. D609	0.07422	***	0.0002
Ctr vs. Manoalide	0.1361	****	<0.0001

e

Dex Ca^{2+} release

Two-Way Anova with Sidak's multiple comparisons test	Mean Diff	Summary	p-value
Nalm-6 cells			
Ctr vs. U73122	0.05474	****	<0.0001
Ctr vs. Edelfosine	0.03574	***	0.0004
Ctr vs. 3-Nitrocoumarin	0.04252	****	<0.0001
Ctr vs. SPK-601	0.02679	**	0.0092
Ctr vs. D609	0.03465	***	0.0007
Ctr vs. Manoalide	0.03479	***	0.0006
RS4;11 cells			
Ctr vs. U73122	0.02350	*	0.0267
Ctr vs. Edelfosine	0.04662	****	<0.0001
Ctr vs. 3-Nitrocoumarin	0.05283	****	<0.0001
Ctr vs. SPK-601	0.08714	****	<0.0001
Ctr vs. D609	0.06334	****	<0.0001
Ctr vs. Manoalide	0.08153	****	<0.0001

Supplementary Figure 8. Dex activates Ca^{2+} signaling via PLC pathway in B-ALL cells.

(**a**) Representative flow cytometry plots of the active form of PLCγ2 (phospho-PLCγ2 (Tyr759)) stimulated with Dex or Ctr for 5 min. (**b**) Quantification from panel (**a**) of the active form of PLCγ2 (phospho-PLCγ2 (Tyr759)) stimulated with Dex or Ctr for 5 min and normalized to Ctr. Data are mean ± SEM from n=3 independent experiments. Statistical analysis was conducted by two-tailed, unpaired Student's t test. (**c**) Representative traces of ER Ca^{2+} release in RS4;11 cells stimulated with 125 nM Dex in Ca^{2+}-free buffer (0 mM Ca^{2+}). Cells were preincubated with 10 µM Edelfosine during Fura-2 loading before stimulation. (**d** and **e**) P-values based on two-way ANOVA with Sidak's multiple comparisons test related to Figure 4**j** and 4**m**, respectively. Source data are provided as a Source Data file.

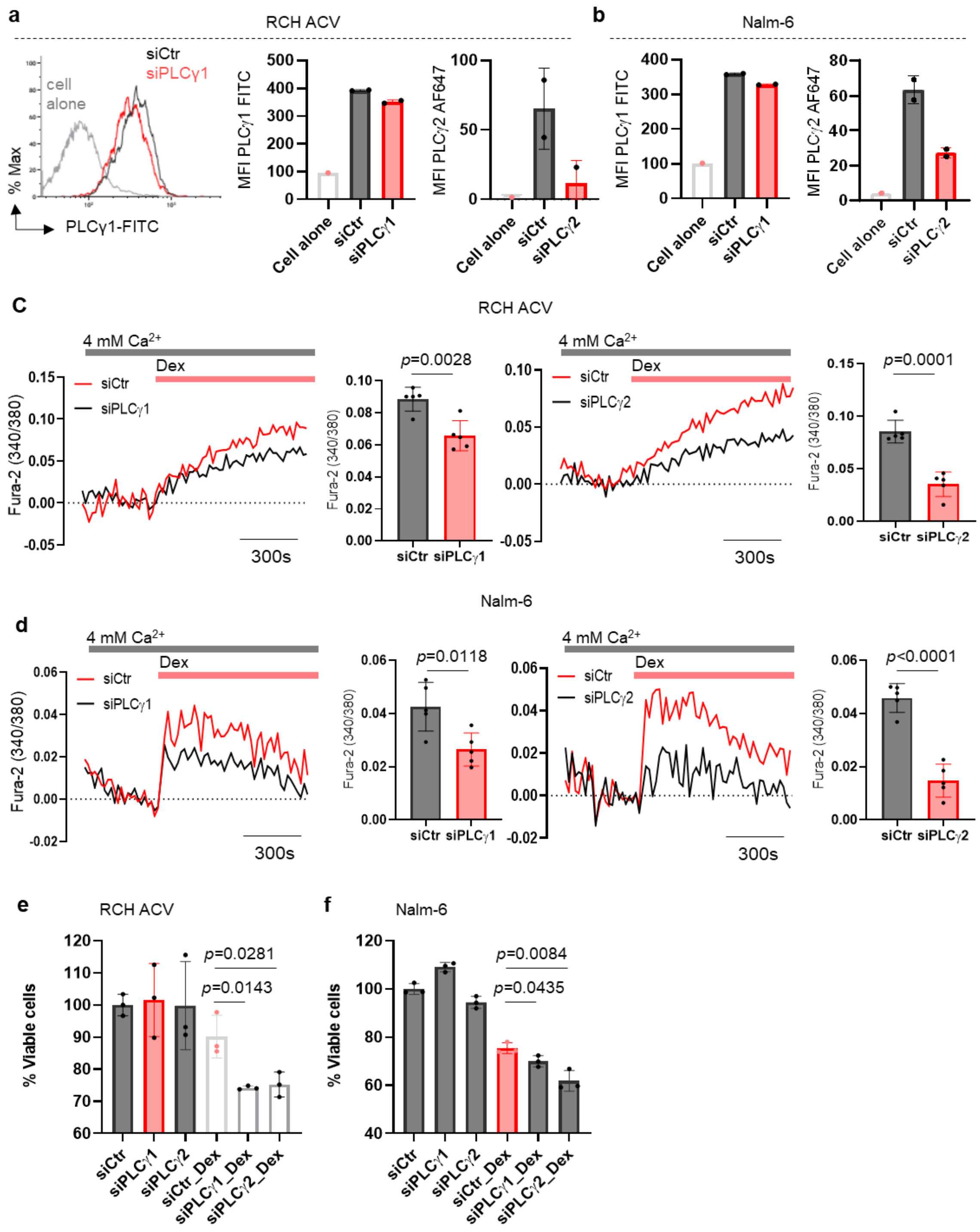

Supplementary Figure 9. Silencing PLCγ1 and PLCγ2 enhances Dex sensitivity in B-ALL cells.

(**a,b**) Representative flow cytometry plots of PLCγ1 (left) and mean fluorescence intensity (MFI, right) of PLCγ1 and PLCγ2 in RCH ACV and Nalm-6 cells transfected with either siCtr or siPLCγ1 and siPLCγ2. Data are presented as means ± SEM (n=2 independent experiments). (**c,d**) Cytosolic Ca^{2+} measurements in RCH-ACV (**c**) and Nalm-6 (**d**)cells transfected with either siCtr or siPLCγ1 and siPLCγ2 then stimulated with 125 nM Dex in nominally Ca^{2+}-containing buffer (4 mM Ca^{2+}). Data are presented as means ± SEM (n=5 independent experiments). Statistical significance was analyzed by two-tailed, unpaired Student's *t* test. (**e,f**) Cell viability was determined by CCk-8 staining after 48 hours of treatment. The percentage of viable cells was established after normalizing cells on Ctr cells. Data are presented as means ± SEM (n=3 independent experiments). Statistical significance was analyzed by two-tailed, unpaired Student's *t* test. Source data are provided as a Source Data file.

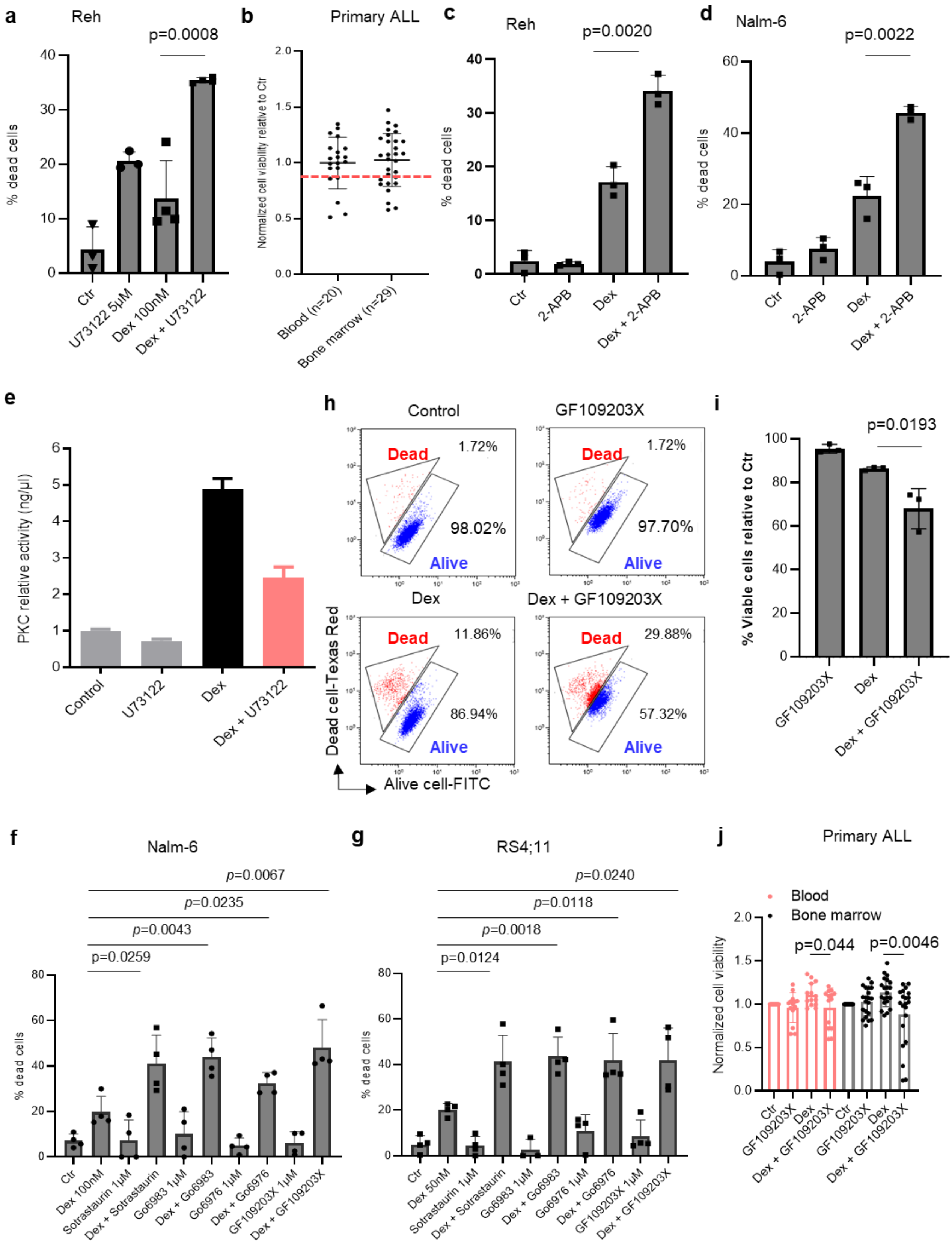

Supplementary Figure 10. PKC Inhibition enhances Dex sensitivity in B-ALL cells.

(**a**) Reh cell mortality was determined by CCk-8 staining after 48 hours of treatment. Data are represented as mean ± SEM (n=3 for Ctr & U73122 and n=4 for Dex and Dex+U73122; n-values correspond to independent experiments). Two-tailed, unpaired Student's *t* test. (**b**) Viability relative to the Ctr of cells from

44 primary ALL samples (at time of diagnosis) exposed to 100 nM Dex for 48 hours. The red line indicates the viability cutoff used to define Dex resistance. (**c and d**) Reh (**c**) and Nalm-6 (**d**) cells treated with 100 nM Dex and 5µM 2-APB alone or in combination. Cell mortality was determined by CCk-8 staining after 48 hours of treatment. Data are presented as means ± SEM (n=3 independent experiments). Two-tailed, unpaired Student's *t* test. (**e**) Quantification of PKC activity in Nalm-6 cells stimulated with 100 nM Dex or Ctr for 24 hours with or without 1 µM U73122 and normalized to Ctr). Data are mean ± SEM from n=2 independent experiments, performed in technical triplicate and measured by ELISA. Two-tailed, unpaired Student's *t* test. (**f** and **g**) Nalm-6 (**f**) and RS4;11 (**g**) cells treated with Dex, Sotrastaurin, Go6983, Go6976 and GF109203X alone or in combination. Cell mortality was determined by CCk-8 staining after 48 hours of treatment. The percentage of dead cells was established after normalizing cells on Ctr cells. Two-tailed, unpaired Student's *t* test; Data shown are the mean ± SEM from n = 4 independent experiments. (**h**) Nalm-6 cells treated with 100nM Dex and 1 µM GF109203X alone or in combination for 48 hours of treatment. Alive and dead cells were determined by flow cytometry after LIVE/DEAD staining. (**i**) Quantification of viable cell populations from panel (**h**), results are presented as means ± SEM (n=3 independent experiments), two-tailed, unpaired Student's t test. (**j**) Viability of cells from the 35 Dex-resistant primary diagnostic ALL samples (panel **b**) exposed to 100 nM Dex and/or 1µM GF109203X for 24 hours, normalized to Ctr condition, two-tailed, unpaired t test. Diagnostic samples of ALL were collected from the peripheral blood (n=14 patients) and bone marrow (n=21 patients) of the patients. Data are mean ± SEM. Source data are provided as a Source Data file.

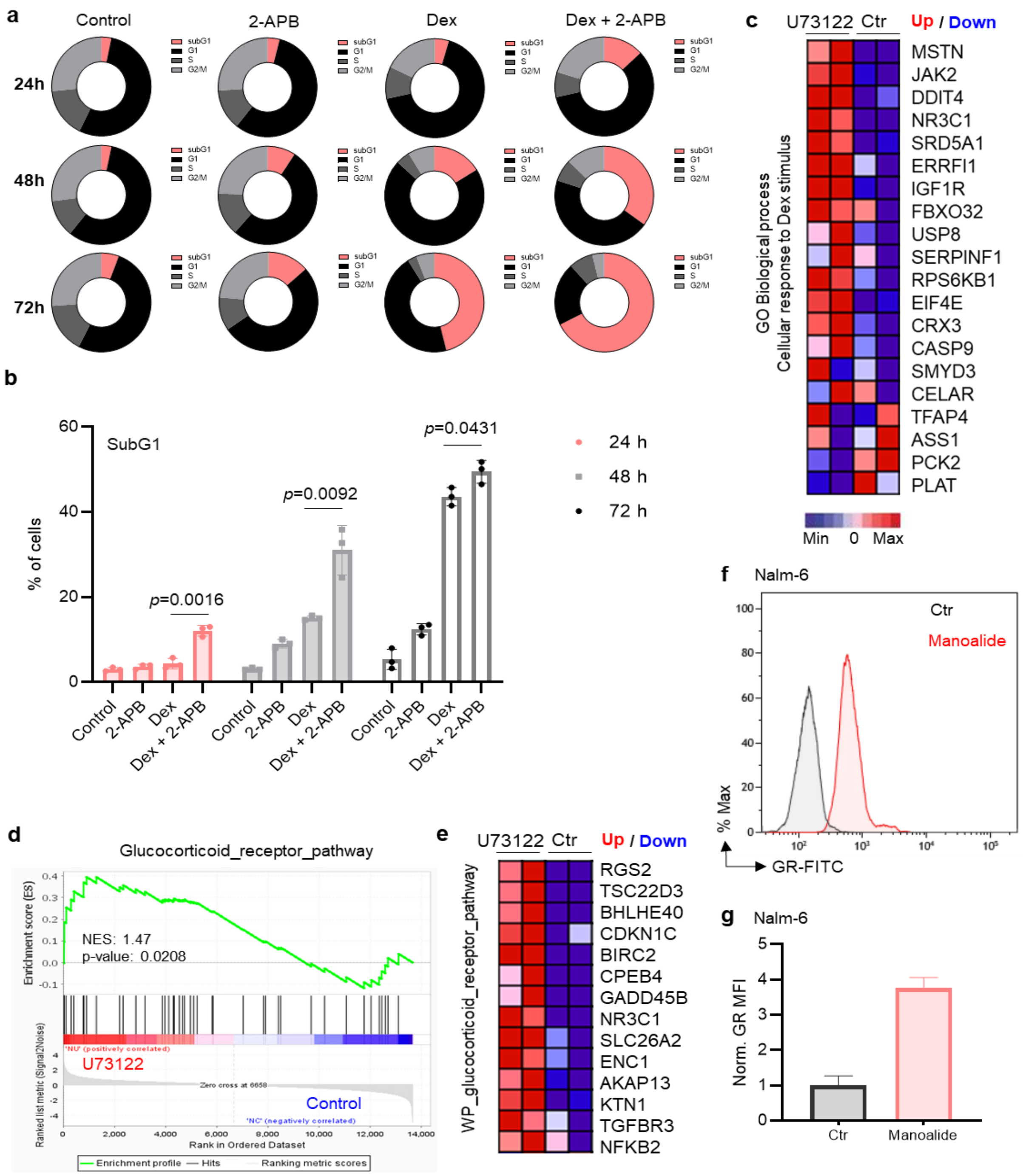

Supplementary Figure 11. PLC inhibition enhances cellular response to Dex stimulus in B-ALL cells.

(**a**,**b**) Cell cycle analysis of Nalm-6 cells before and after stimulation with 100nM Dex in the presence or absence of 5 µM 2-APB for 24, 48 and 72 hours. Distribution (**a**) of the different cell cycle phases, and quantification (**b**) of subG1 cell cycle phases from panel **a.** Data are mean ± SEM from n=3 independent experiments in (**b**). (**c**) Differentially expressed genes between U73122- or Ctr-treated Nalm-6 R cells and heatmap demonstrating the different expression profiles of the cellular response to Dex stimulus from Biological process (n=2 biological replicates). (**d**) Representative GSEA enrichment plot demonstrating the upregulation of glucocorticoid receptor pathway-related genes in U73122-treated Nalm-6 R cells versus Ctr (n=2 biological replicates). (**e**) Heatmap demonstrating the different expression profiles of the glucocorticoid receptor pathway from panel D (n=2 biological replicates). (**f**) Representative flow cytometry plots of glucocorticoid receptor (GR) stimulated with PLC inhibitor for 24 hours. (**g**) Quantification of the GR from panel (**f**) and normalized to Ctr. Data are mean ± SEM from n=2 independent

experiments. Statistical analysis was conducted by two-tailed, unpaired Student's t test. Source data are provided as a Source Data file.

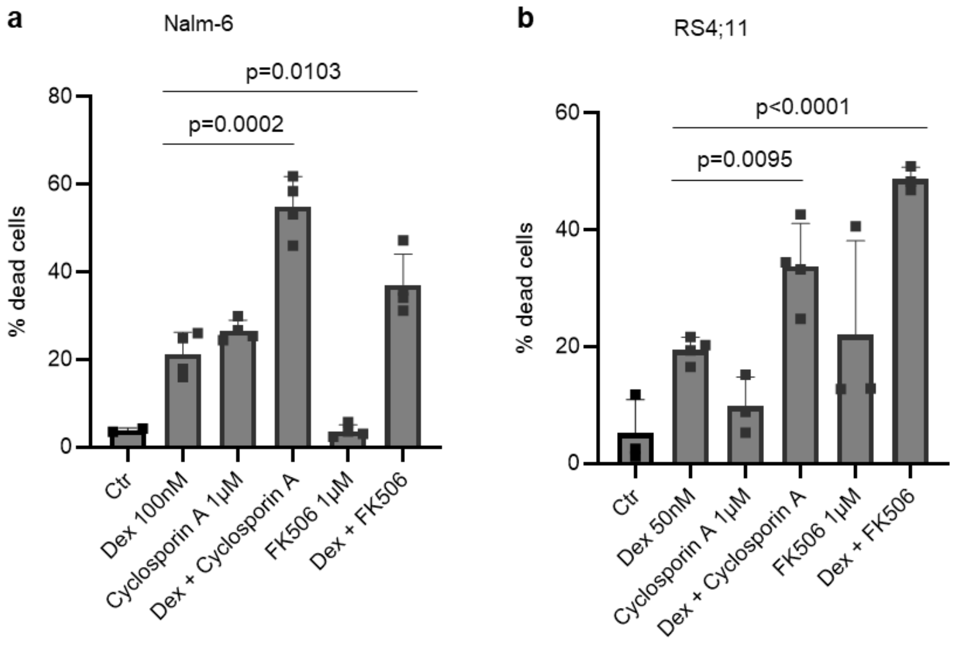

Supplementary Figure 12. Calcineurin inhibition enhances Dex sensitivity in B-ALL cells.

Nalm-6 (**a**) and RS4;11 (**b**) cells treated with Dex and calcineurin inhibitors alone or in combination. Cell mortality was determined by CCk-8 staining after 48 hours of treatment. The percentage of dead cells was established after normalizing cells on Ctr cells. Data are represented as mean ± SEM, representative of n=3 independent experiments. Statistical analysis was conducted by two-tailed, unpaired Student's t test. Source data are provided as a Source Data file.

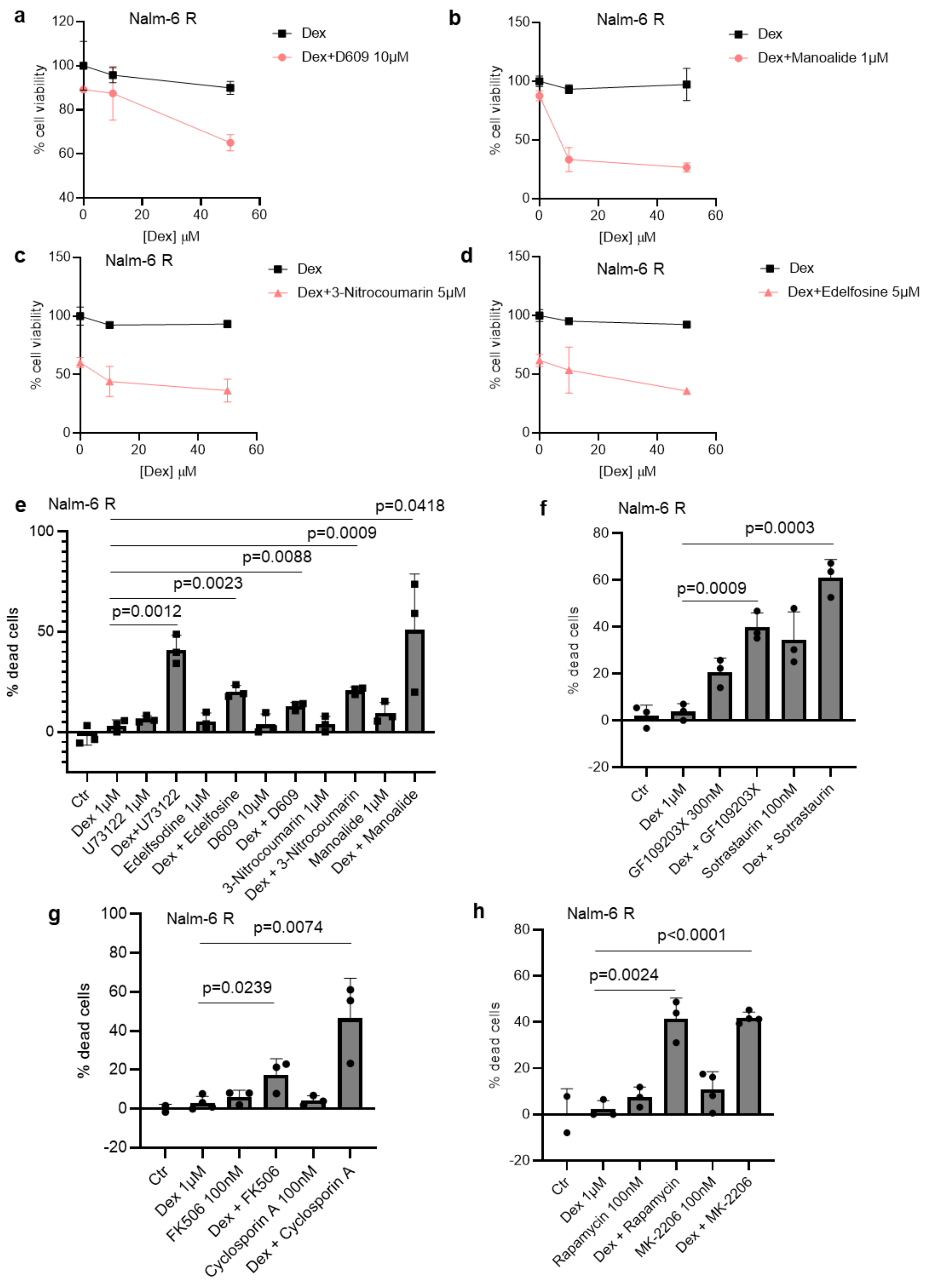

Supplementary Figure 13. PLC, PKC, calcineurin, mTOR or AKT inhibition reversed Dex resistance in Dex- resistant B-ALL cells.

(**a-d**) Viability relative to the Ctr of Dex-resistant ALL cells exposed for 48 hours with increasing concentrations of Dex with or without PLC inhibitors. Data are representative of n=2 independent experiments. (**e**) Dex- resistant ALL cells treated with Dex, U73122, Edelfosine, 3-Nitrocoumarin, D609 and Manoalide alone or in combination. Cell mortality was determined by CCk-8 staining after 48 hours of

treatment. The percentage of dead cells was established after normalizing cells on Ctr cells. Two-tailed, unpaired Student's t test; data are presented as means ± SEM of n=3 independent experiments. (**f**) Dex-resistant ALL cells treated with Dex, Sotrastaurin and GF109203X alone or in combination. Cell mortality was determined by CCk-8 staining after 48 hours of treatment. The percentage of dead cells was established after normalizing cells on Ctr cells. Two-tailed, unpaired Student's t test; data are presented as means ± SEM of n=3 independent experiments. (**g,h**) Dex-resistant ALL cells treated with Dex, Cyclosporin A and FK506 alone or in combination (**g**) and with Dex, Rapamycin and MK-2206 alone or in combination (**h**). Cell mortality was determined by CCk-8 staining after 48 hours of treatment. Two-tailed, unpaired Student's t test. Data are represented as mean ± SEM, representative of n=3 independent experiments. Source data are provided as a Source Data file.

a

Gene set	Gene name
Genes strongly correlated (r>0,5) with PLCγ2 in GSE13159<br><br>n = 281	QSOX2 TAGAP PPARD SNN IRF4 RRAS2 CLNK UGCG RAB30 GABBR1 STK10 APBB2 OSBPL10 TAGLN IL16 INPP5F SPIB TMEM243 TBX21 BLNK FAM65A RALGPS1 NCOA3 HLA-F FAM3C PPFIBP2 IGHD CD37 IL4R FAM214A KIAA0226L CCDC69 SLC9A7 PARP15 SIDT1 TEAD2 IGHM MALT1 BMF WFDC21P KLHL14 RASSF3 PCED1B TGFBR2 RAPGEF1 BTLA TTN ANKRD13A HLA-DMA CAPN3 CDK14 SP140L NLRP1 MKNK2 MS4A1 CYTH1 PTPN1 WDR91 TGFBR3 HLA-DPA1 HLA-DRB1 MTSS1 WDR11 BANK1 CREBBP VCPKMT RNF41 FAM53B CLEC2D LSR DUS2 ENDOU ALOX5 SAMD8 UVRAG FCER2 HLA-DOA CCDC50 HLA-DMB PHLPP2 ARHGAP17 HMHA1 LAPTM5 LYN FAM129C NAPSB PTPN12 SLC25A28 PRKCB MAP3K5 SP100 SLC23A2 TRAK1 ZMIZ2 SYT17 DENND4B COL9A2 SLC44A2 FOXP1 RRM2B TRIM26 MAML2 LRRK1 CHD7 CPNE5 ARHGAP25 UNC93B1 C18ORF8 WIPF2 SYNGR3 PRDM2 SEL1L3 ZNF274 NLRC5 TFEB STIM2 FCRL2 NFKBID CCDC191 TCP11L2 KIAA1551 KMO SIPA1L3 ABCA6 ENTHD2 AKT2 AIM2 IRF8 LOC100289090 KIAA0430 ABR DNMBP LOC100505564 TCL1A ZC3H12D FARP2 CLIP1 PLEKHG1 MARCH3 VOPP1 FCRLA CD79A HLA-DOB ZNF791 LOC728392 WDFY4 CTLA4 RASGRF1 ST6GAL1 HLA-E C14ORF28 SWAP70 LY9 GRB2 TMEM55B NDUFV2-AS1 CHST15 SETX MCM9 SFMBT1 LMBRD1 TBC1D1 FMOD ARHGAP24 IFT57 ENTPD4 LOC374443 MGAT5 ZNF75A ZNF266 DOCK10 TNFRSF10A SPOCK2 FAM159A MAP3K14 PHC3 MATN1-AS1 LINC-PINT SH2B2 PLCL2 HLA-DPB1 ICOSLG CD74 CLLU1 ADAM19 FCHSD2 HLA-DRA SERPINB9P1 CYB561A3 SETBP1 SPOPL SLC6A16 GEMIN7 CBX7 CD40 CD19 HVCN1 RAPGEF3 SNX25 DEPDC5 GPR18 BACH2 FCMR ANKRD44 TBC1D9 GSAP ROR1 STK4 LINC00494 SEMA4B PIGR KIAA0355 CGRRF1 PATL2 CELSR1 ZNF318 FCRL3 IKZF3 TRAPPC10 ZBP1 EZR WNT3 TNFRSF13C ARID5B MAP3K1 SMAP2 PHTF2 ISG20 ZBTB32 RALGPS2 ABCB4 FCRL1 PAX5 LINC00926 MOB1A POU2F2 ZBTB4 CAPN12 WDFY1 PRDM4 CIITA FOXO1 BLK GPM6A EVI5 SIDT2 TRAF1 GGA2 MCOLN2 PIM2 ZNF600 GYLTL1B PNOC LRMP MAP2 TMEM62 TCF4 P2RY10 YPEL3 HLA-DQB1 RUBCN CHMP7 REM2 IFNLR1 MAST3 YPEL2 ARHGAP44 ADTRP FGD2 PDE4B FCRL5 BCAS4 RBM38 RERE EBLN3 ZNF831 STAP1 KIAA0040 CHKB KHNYN PLEKHA2

b

Gene set	Gene name
GOBP_Calcium_ mediated_Signaling<br><br>n = 197	CALM3 RIT2 PLCE1 MTOR ZAP70 ADGRL1 SLA2 EDN1 ACTN3 CASQ1 CACNA1C CCR10 TMBIM4 NMUR1 IGF1 SELENOK VCAM1 KDR ADGRB2 TNF CHP1 ACKR4 RGN NCALD P2RX5 CIB1 HOMER3 CCR8 CCR2 CD4 NMUR2 ANK2 PTK2B GPR143 CCR6 CHP2 FHL2 PLCG2 SELP PLEK PPP3CB CDH13 MYOZ2 CXCR1 HTR2B CCR1 RCAN1 ATP1A2 GSTO1 TRPM8 P2RX4 EDNRB HRC CHERP NFATC4 NEUROD2 GRIN2B DEFB1 PDPK1 PTPRJ PDK2 DMD TPCN2 CCR5 SPHK1 CCL20 FPR2 PRKACA NFATC1 CCR9 HOMER2 SLC8A1 PLN SLC8A2 MCOLN1 CAMKK2 STIMATE PPP3R1 ITPR1 PPP3CA EDN2 TREM2 TMEM38A PLCG1 KSR2 TNFSF11 NUDT4 LMCD1 EGFR FIS1 P2RY12 CHRM3 CXCR6 DMTN CXCR3 SAMD14 SULT1A4 AKAP6 IRGM EFHB C10orf71 CCL4 TRPM2 HTT POMC ATP1B1 ERBB3 ZMPSTE24 LAT2 SYK MAPK7 GRIN2D NFAT5 AGTR1 GRIN1 CLEC7A LAT CMKLR1 P2RX3 SPPL3 MCU NFATC3 GBP1 CAMTA1 TBC1D10C CASQ2 GRM5 CLIC2 HPCA P2RY11 CX3CR1 RCAN3 MYOZ1 PPP1R9A PLA2G4B FKBP1B P2RX7 SULT1A3 NR5A2 AVPR1A GRIN2A ACKR2 CXCR2 CAMK2D CCL3 BCAP31 GSTM2 MCTP2 TNNI3 ATP2A2 NRG1 LRRK2 TMEM38B CALM1 HINT1 SLC24A4 BHLHA15 AZU1 RCAN2 CALM2 CD3E PPP1R9B MIR133A1 GSK3B GRIN2C DMPK LACRT RYR2 PRNP ADA EIF2AK3 P2RX2 ATP2B4 MAPT PRKAA1 CCR3 TRPM5 PTBP1 CXCL8 PTGDR2 NFATC2 BST1 PTGFR SELE ACKR3 TMEM100 TRPM4 MIR1-1 TRAT1 XCR1 MCTP1 CCRL2 DYRK2 PTPRC CCR4 SGCD BTK

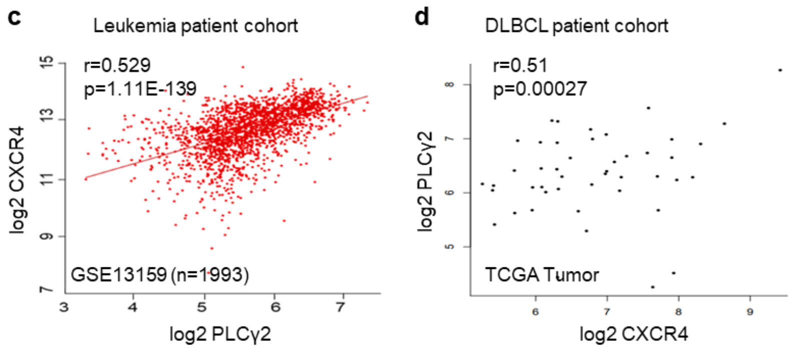

Supplementary Figure 14. PLCγ2 expression is strongly correlated with CXCR4 expression in leukemia.

(**a**) List of genes strongly correlated (Pearson's *r* >0.5) with PLCγ2 in MILE Leukemia from R2. (**b**) List of calcium mediated signaling genes from MSigDB GO biological process set. (**c**,**d**) Positive correlation between active PLCγ2 and CXCR4 in leukemia patient cohort (Pearson's *r* = 0.529, p=1.11E-139) (**c**) and in DLBCL patient cohort (Pearson's *r* = 0.51, p=0.00027) (**d**). Data were extracted from the R2 and Gene Expression Profiling Interactive Analysis (GEPIA), respectively. Source data are provided as a Source Data file.

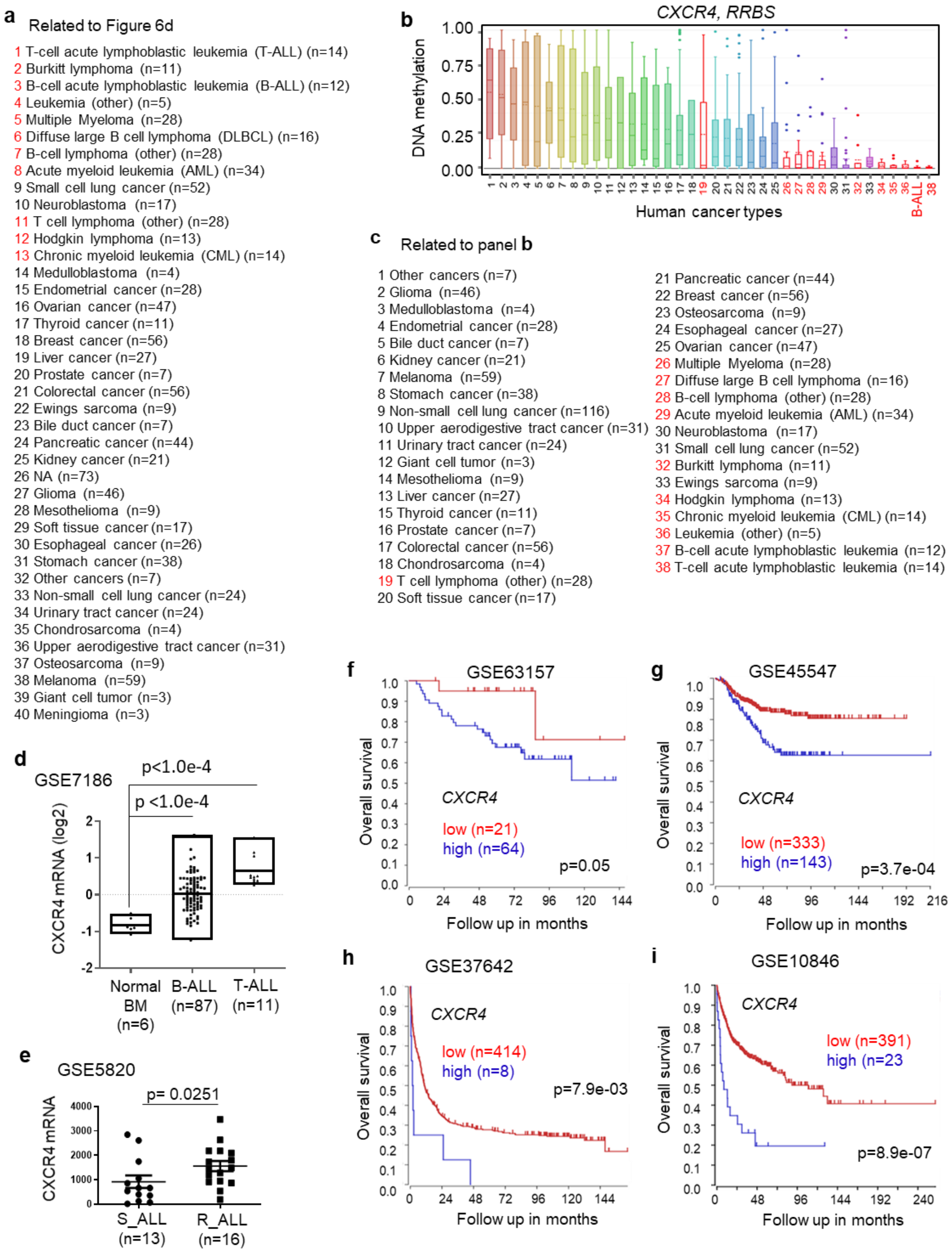

Supplementary Figure 15. High CXCR4 expression is correlated with poor patient survival in several pediatric cancers.

(**a**) Human cancer cell lines from the CCLE database, ranked by CXCR4 mRNA expression (high to low). Related to Figure 7d. Numbers are the same as those used in the graph in Figure 7d. Hematological malignancies are in red. 'n' represents the number of cell lines analyzed in each cancer type. (**b**,**c**) CXCR4

mRNA ranked by expression level in B-ALL and other human tumors (**b**). Data were extracted from the CCLE. Numbers refer to cancer cell types and hematological malignancies are in red (**c**). 'n' in (**c**) represents the number of cell lines analyzed in each cancer type. (**d**) Publicly available transcriptomic dataset analysis showed the expression levels of CXCR4 across B-ALL, T-ALL and normal cells. Statistical analysis was conducted by two-tailed, unpaired Student's t test. (**e**) CXCR4 mRNA expression between sensitive (S) and resistant (R) ALL in publicly available transcriptomic dataset. Statistical analysis was conducted by two-tailed, unpaired Mann Whitney test. (**f-i**) Kaplan–Meier analysis with median cutoff values of CXCR4 expression and overall survival in Ewing sarcoma – Dirksen (**f**), neuroblastoma - Kocak (**g**), acute myeloid leukemia-Bohlander (**h**), B-cell lymphoma-Xiao (**i**). Data were extracted from R2 database. P-values were calculated by the log–rank test. Source data are provided as a Source Data file.

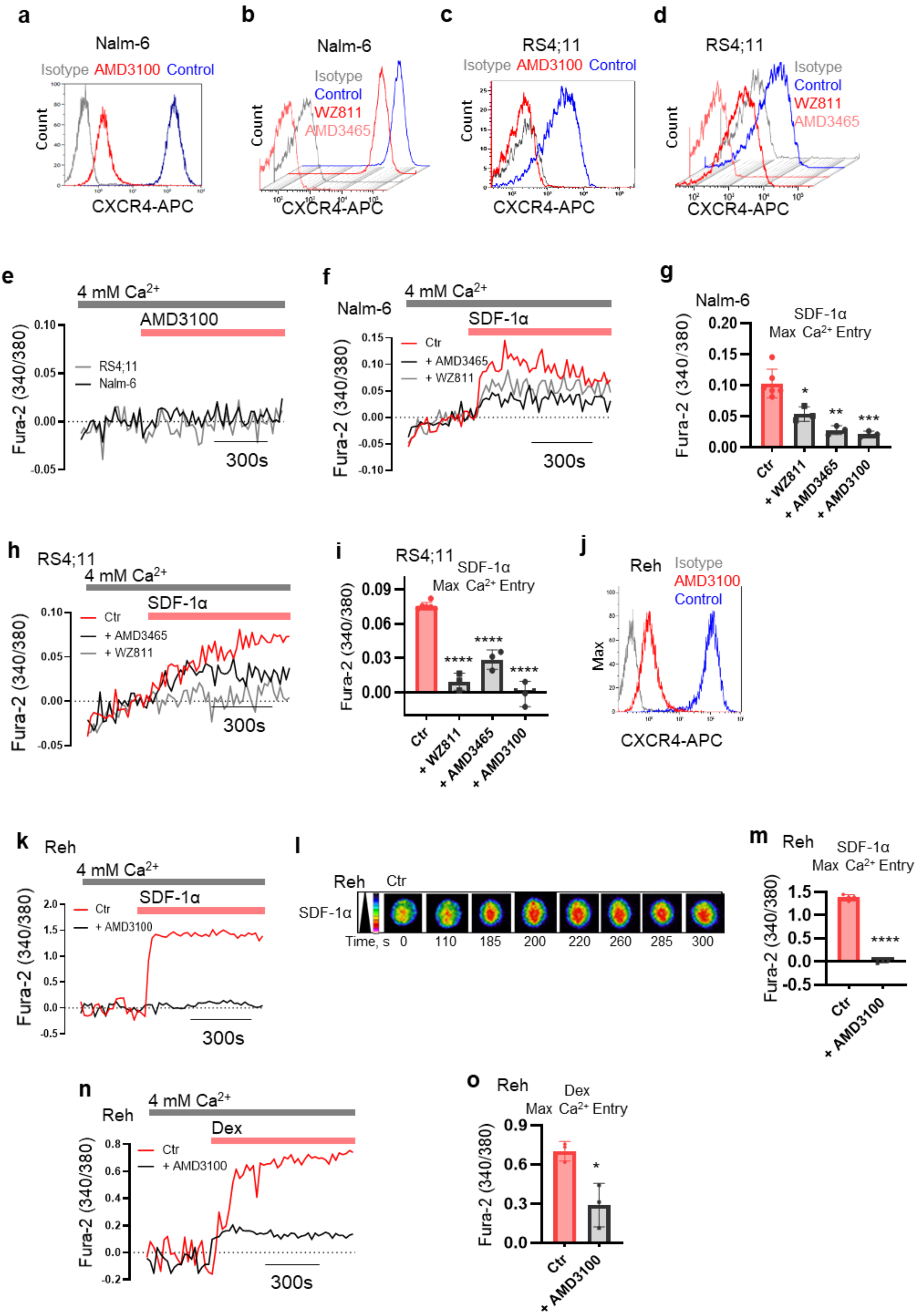

a
Nalm-6
Isotype AMD3100 Control
Count
CXCR4-APC
b
Nalm-6
Isotype
Control
WZ811
AMD3465
Count
CXCR4-APC
c
RS4;11
Isotype AMD3100 Control
Count
CXCR4-APC
d
RS4;11
Isotype
Control
WZ811
AMD3465
Count
CXCR4-APC
e
4 mM Ca2+
AMD3100
RS4;11
Nalm-6
Fura-2 (340/380)
300s
f
Nalm-6
4 mM Ca2+
SDF-1α
Ctr
+ AMD3465
+ WZ811
Fura-2 (340/380)
300s
g
Nalm-6
SDF-1α
Max Ca2+ Entry
Fura-2 (340/380)
Ctr
+ WZ811
+ AMD3465
+ AMD3100
h
RS4;11
4 mM Ca2+
SDF-1α
Ctr
+ AMD3465
+ WZ811
Fura-2 (340/380)
300s
i
RS4;11
SDF-1α
Max Ca2+ Entry
Fura-2 (340/380)
Ctr
+ WZ811
+ AMD3465
+ AMD3100
j
Reh
Isotype
AMD3100
Control
Max
CXCR4-APC
k
Reh
4 mM Ca2+
SDF-1α
Ctr
+ AMD3100
Fura-2 (340/380)
300s
l
Reh
Ctr
SDF-1α
Time, s 0 110 185 200 220 260 285 300
m
Reh
SDF-1α
Max Ca2+ Entry
Fura-2 (340/380)
Ctr
+ AMD3100
n
Reh
4 mM Ca2+
Dex
Ctr
+ AMD3100
Fura-2 (340/380)
300s
o
Reh
Dex
Max Ca2+ Entry
Fura-2 (340/380)
Ctr
+ AMD3100

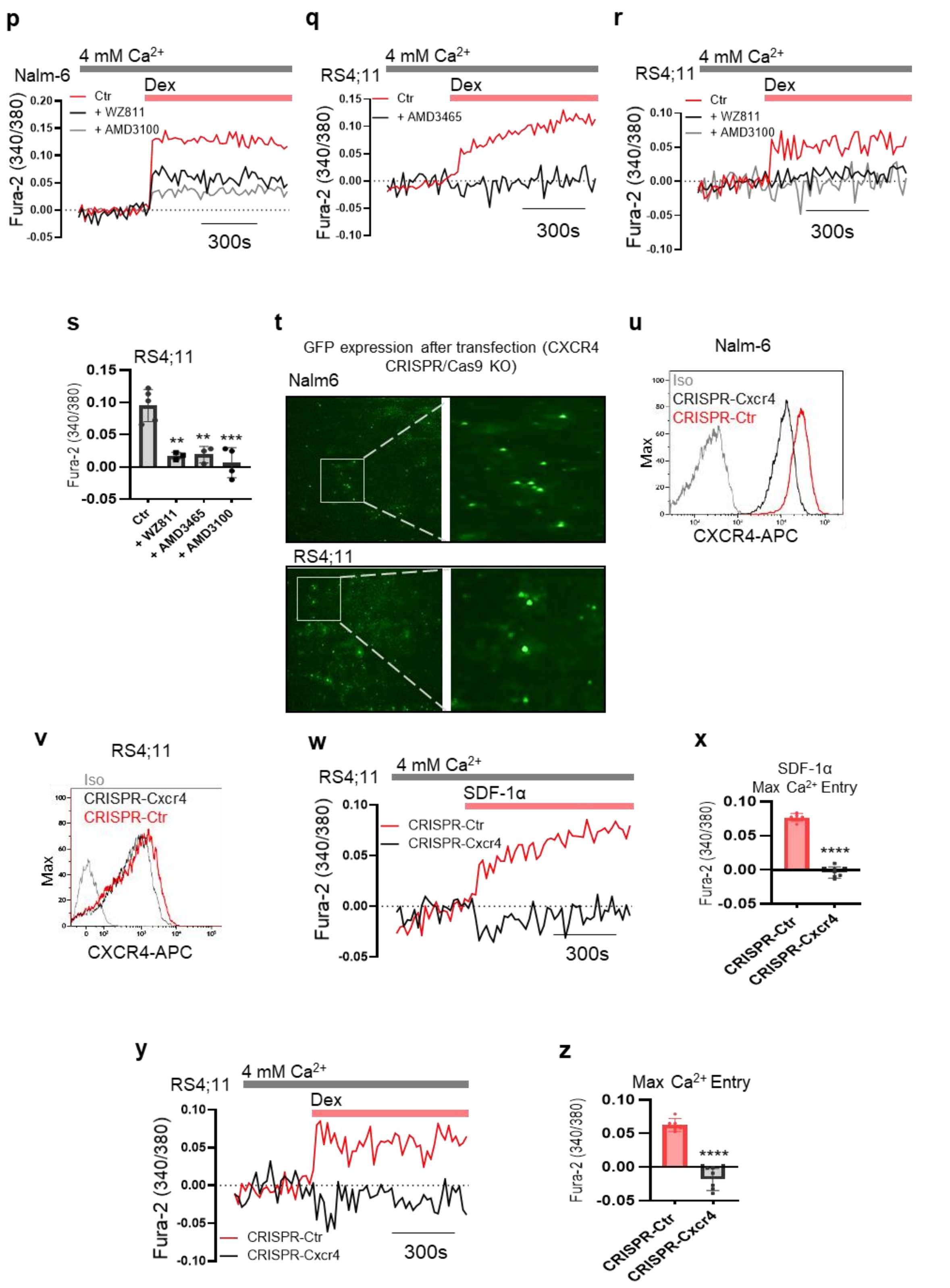

Continued...

Supplementary Figure 16. CXCR4 mediates SDF-1α- and Dex-induced Ca^{2+} signaling in ALL cells.

(**a-d**) Representative flow cytometry plots of CXCR4 surface expression in Nalm-6 cells (**a**,**b**) and RS4;11 cells (**c**,**d**) treated with CXCR4 antagonists, AMD3100, AMD3465 and WZ811. (**e**) Averaged cytosolic Ca^{2+} measurements in Nalm-6 and RS4;11 cells stimulated with 25 μM AMD3100. Data are mean ± SEM (n=3

independent experiments). (**f**) Averaged cytosolic Ca^{2+} measurements in Nalm-6 cells stimulated with 100 nM SDF-1α in the presence or absence of CXCR4 antagonists. Data are mean ± SEM (n=3 independent experiments). (**g**) Quantification of maximal Ca^{2+} entry of Nalm-6 cells stimulated with 100 nM SDF-1α in the absence or presence of three CXCR4 antagonists (WZ811, AMD3465 or AMD3100). Data are the mean ± SEM from (n = 6 for Ctr and n=3 for antagonists; n-values correspond to independent experiments). (**h**) Cytosolic Ca^{2+} measurements in RS4;11 cells stimulated with 100 nM SDF-1α in the presence or absence of CXCR4 antagonists. Data are mean ± SEM (n=3 independent experiments). (**i**) Quantification of maximal Ca^{2+} entry of RS4;11 cells stimulated with 100 nM SDF-1α in the presence or absence of three CXCR4 antagonists (WZ811, AMD3465 or AMD3100). Data are the mean ± SEM from (n = 6 for Ctr and n=3 for antagonists; n-values correspond to independent experiments). (**j**) Representative flow cytometry plots of CXCR4 surface expression in Reh cells treated with CXCR4 antagonist, AMD3100. (**k**) Cytosolic Ca^{2+} measurements in Reh cells stimulated with 100 nM SDF-1α in the presence or absence of CXCR4 antagonist. Data are mean ± SEM (n=4 independent experiments). (**l**) Colored time-lapse images of Reh cells show the changes in cytosolic Ca^{2+} evoked by SDF-1α in Ca^{2+}-containing buffer. (**m**) Quantification of maximal Ca^{2+} entry of Reh cells stimulated with 100 nM SDF-1α in the presence or absence of CXCR4 antagonist. Data are mean ± SEM (n=4 independent experiments). (**n**) Cytosolic Ca^{2+} measurements in Reh cells stimulated with 125 nM Dex in the presence or absence of CXCR4 antagonist. Data are mean ± SEM (n=3 independent experiments). (**o**) Quantification of maximal Ca^{2+} entry of Reh cells stimulated with 125 nM Dex in the presence or absence of CXCR4 antagonist. Data are mean ± SEM (n=3 independent experiments). (**p**) Cytosolic Ca^{2+} measurements in Nalm-6 cells stimulated with 125 nM Dex in the presence or absence of CXCR4 antagonists. Data are mean ± SEM (n=3 independent experiments). (**q** and **r**) Cytosolic Ca^{2+} measurements in RS4;11 cells stimulated with 125 nM Dex in the presence or absence of CXCR4 antagonists. Data are mean ± SEM (n=3 independent experiments). (**s**) Quantification of maximal Ca^{2+} entry of RS4;11 cells stimulated with 125 nM Dex in the presence or absence of CXCR4 antagonists. Data are the mean ± SEM from (n = 5 for Ctr, n=3 for WZ811 & AMD3465 and n=4 for AMD3100; n-values correspond to independent experiments). (**t**) Detection of green fluorescent protein (GFP) after successful transfection of the CXCR4CRISPR/Cas9 KO plasmid in Nalm-6 cells. (**u**) Representative flow cytometry plots of CXCR4 surface expression in Nalm-6 cells transduced with CRISPR-Ctr or CRISPR-Cxcr4. (**v**) Representative flow cytometry plots of Cxcr4 surface expression in RS4;11 cells transduced with CRISPR-Ctr or CRISPR-CXCR4 . (**w**) Cytosolic Ca^{2+} measurements in RS4;11 cells transduced with either CRISPR-Ctr or CRISPR-CXCR4 then stimulated with 100 nM SDF-1α in nominally Ca^{2+}-containing buffer (4 mM Ca^{2+}). Data are mean ± SEM (n=6 independent experiments). (**x**) Quantification of maximal Ca^{2+} entry from panel **w**. Data are mean ± SEM (n=6 independent experiments). (**y**) Cytosolic Ca^{2+} measurements in RS4;11 cells transduced with either CRISPR-Ctr or CRISPR-CXCR4 then stimulated with 125 nM Dex in nominally Ca^{2+}-containing buffer (4 mM Ca^{2+}). Data are mean ± SEM (n=6 independent experiments). (**z**) Quantification of maximal Ca^{2+} entry from panel **y**. Data are mean ± SEM (n=6 independent experiments). All statistical significance was calculated analyzed by two-tailed, unpaired Student's t test. *p < 0.05, **p < 0.01, ***p < 0.001 and ****p < 0.0001. Source data are provided as a Source Data file.

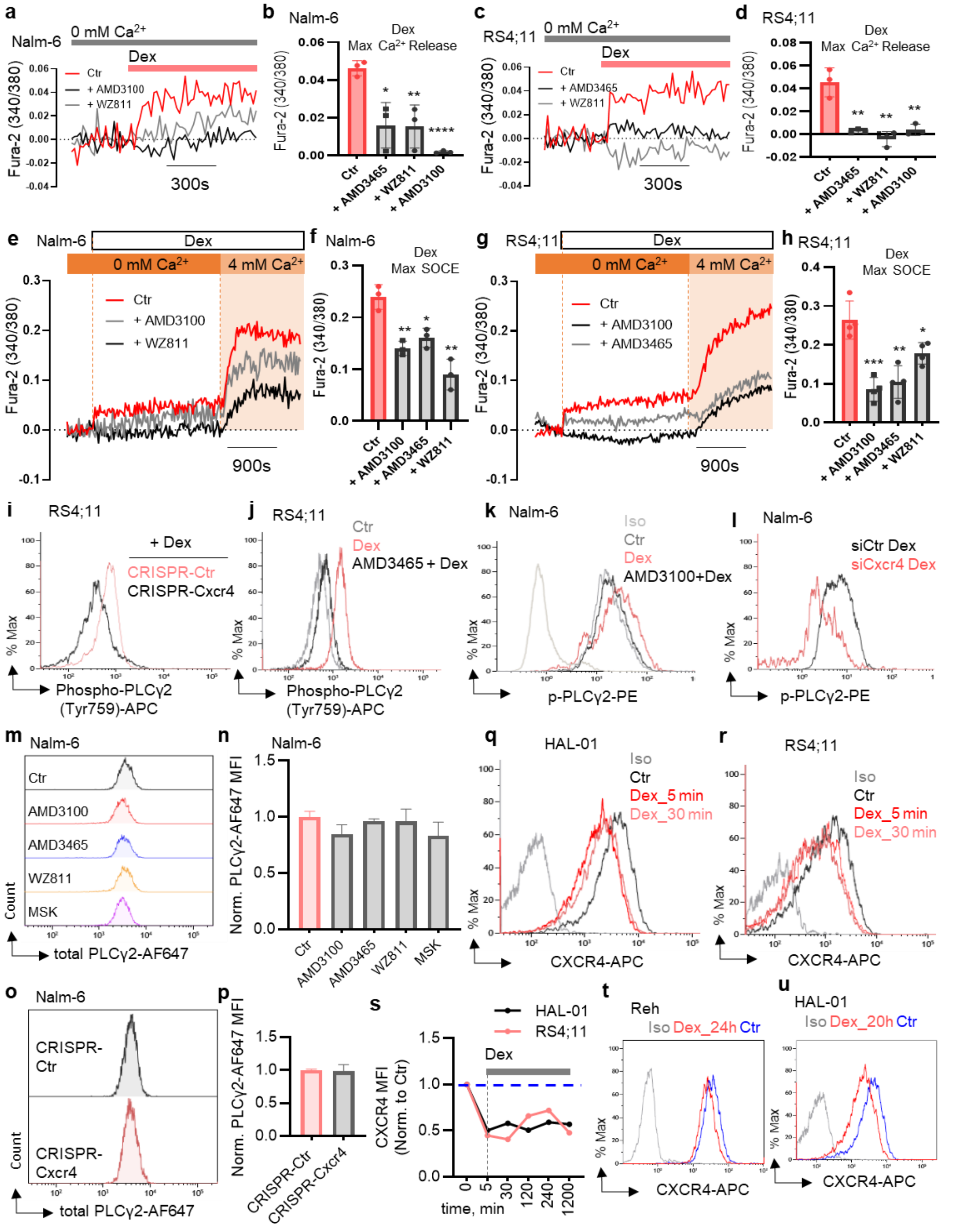

Supplementary Figure 17. Dex activates PLC signaling and CXCR4 internalization in B-ALL cells.

(**a**) ER Ca^{2+} measurements in Nalm-6 cells stimulated with 125 nM Dex in the presence or absence of CXCR4 antagonists in nominally Ca^{2+}-free buffer (0 mM Ca^{2+}). Data are mean ± SEM (n=3 independent experiments). (**b**) Quantification of maximal Ca^{2+} release from panel **a**. Data are mean ± SEM (n=3 independent experiments). (**c**) ER Ca^{2+} measurements in RS4;11 cells stimulated with 125 nM Dex in the presence or absence of CXCR4 antagonists in nominally Ca^{2+}-free buffer (0 mM Ca^{2+}). Data are mean ± SEM (n=3 independent experiments). (**d**) Quantification of maximal Ca^{2+} release from panel **c**. Data are mean ± SEM (n=3 independent experiments). (**e-h**) Cytosolic Ca^{2+} measurements in Nalm-6 (**e**) and RS4;11 (**g**).

Fura-2-loaded cells were stimulated by Dex (125 nM) in Ca^{2+}-free buffer followed by re-addition of extracellular Ca^{2+} (4 mM Ca^{2+} containing buffer). Quantification of SOCE (addition of 4mM Ca^{2+}) in Nalm-6 (**f**) and RS4;11 (**h**) cells. Data are mean ± SEM from n=3 independent experiments. (**i,j**) Representative flow cytometry plots of the active form of PLCγ2 (phospho-PLCγ2 (Tyr759)) induced by 125 nM Dex in RS4;11 cells transfected with either CRISPR-Ctr or CRISPR-CXCR4 (**i**) and in RS4;11 cells in the presence or absence of AMD3465 (**j**). (**k,l**) Representative flow cytometry plots of the active form of PLCγ2 (phospho-PLCγ2 (Tyr759)) induced by 125 nM Dex in Nalm-6 cells in the presence or absence of AMD3100 (**k**) and in Nalm-6 cells transfected with either siCtr or siCXCR4 (**l**). (**m,n**) Representative flow cytometry plots (**m**) and quantification (**n**) of the total form of PLCγ2 stimulated with CXCR4 antagonists or Ctr for 24 hours and normalized to Ctr in Nalm-6 cells. Data are representative of n=2 independent experiments. (**o,p**) Representative flow cytometry plots (**o**) and quantification (**p**) of the total form of PLCγ2 stimulated with CXCR4 antagonists or Ctr for 24 hours and normalized to Ctr in Nalm-6 cells transduced with either CRISPR-Ctr or CRISPR-CXCR4. Data are representative of 2 independent experiments. (**q,r**) Representative flow cytometry plots of CXCR4 surface expression of HAL-01 (**q**) and RS4;11 (**r**) cells stimulated with 125 nM Dex for 5 min and 30 min at 37°C. (**s**) Normalized CXCR4 surface expression of HAL-01 and RS4;11 cells stimulated with 125 nM Dex in time dependent manner at 37°C. Data represented as mean ± SEM (n=2 independent experiments). (**t,u**) Representative flow cytometry plots of CXCR4 surface expression of Reh (**t**) and HAL-01 (**u**) cells stimulated with 125 nM Dex for 24 hours and 20 hours, respectively, at 37°C. The "Ctr" (blue line) and the Iso (grey line) conditions (**u**) were replotted from panel **q**. All statistical significance was analyzed by two-tailed, unpaired Student's t test. *p < 0.05, **p < 0.01, ***p < 0.001 and ****p < 0.0001. Source data are provided as a Source Data file.

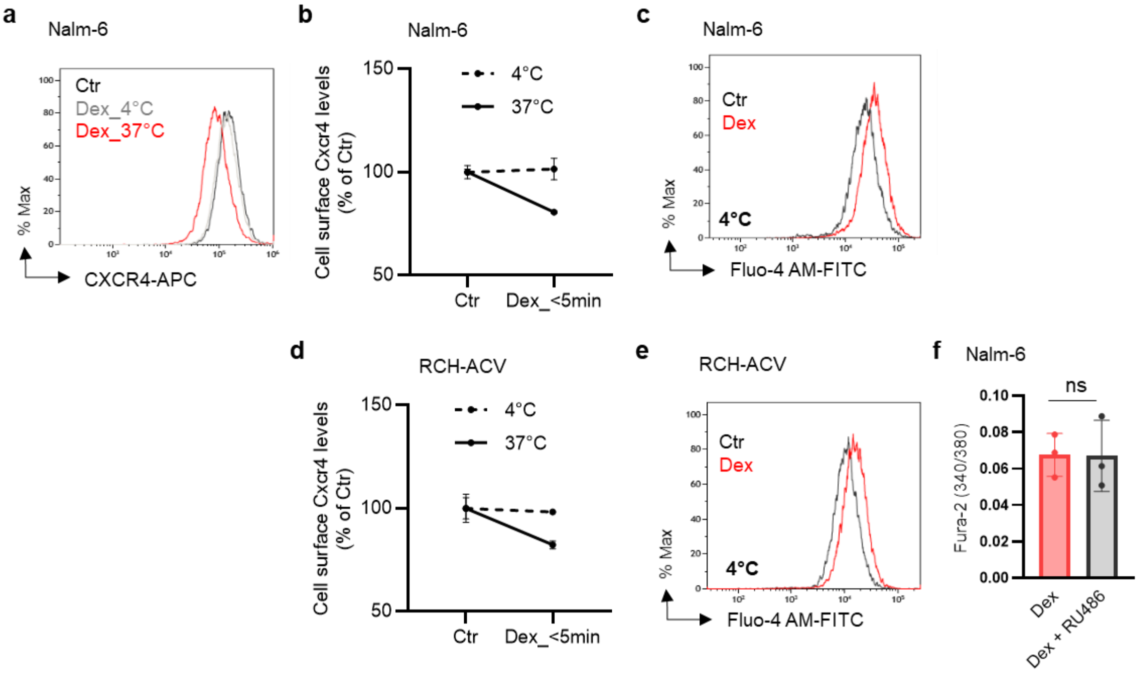

Supplementary Figure 18. Dex-mediated internalization of CXCR4 is a secondary effect of its activation in B-ALL cells

(**a**) Representative flow cytometry plots of CXCR4 surface expression in Nalm-6 cells stimulated with 125 nM Dex for less than 5 min at 4°C and 37°C. (**b,d**) Quantification of CXCR4 surface expression in Nalm-6 cells (**b**) and RCH ACV cells (**d**) stimulated with 125 nM Dex for less than 5 min at 4°C and 37°C. Data are presented as means ± SEM (n=2 independent experiments). (**c,e**) Representative flow cytometry plots of cytosolic Ca^{2+} measurements in Nalm-6 cells (**c**) and RCH ACV cells (**e**) stimulated with 125 nM Dex under the same conditions as in panel (**b,d**). Data are are representative of 2 independent experiments. (**f**) Quantification of cytosolic Ca^{2+} measurements in Nalm-6 cells stimulated with 125 nM Dex in the presence or absence of RU486. Data are mean ± SEM (n=3 independent experiments). Statistical significance was analyzed by two-tailed, unpaired Student's *t* test. Source data are provided as a Source Data file.

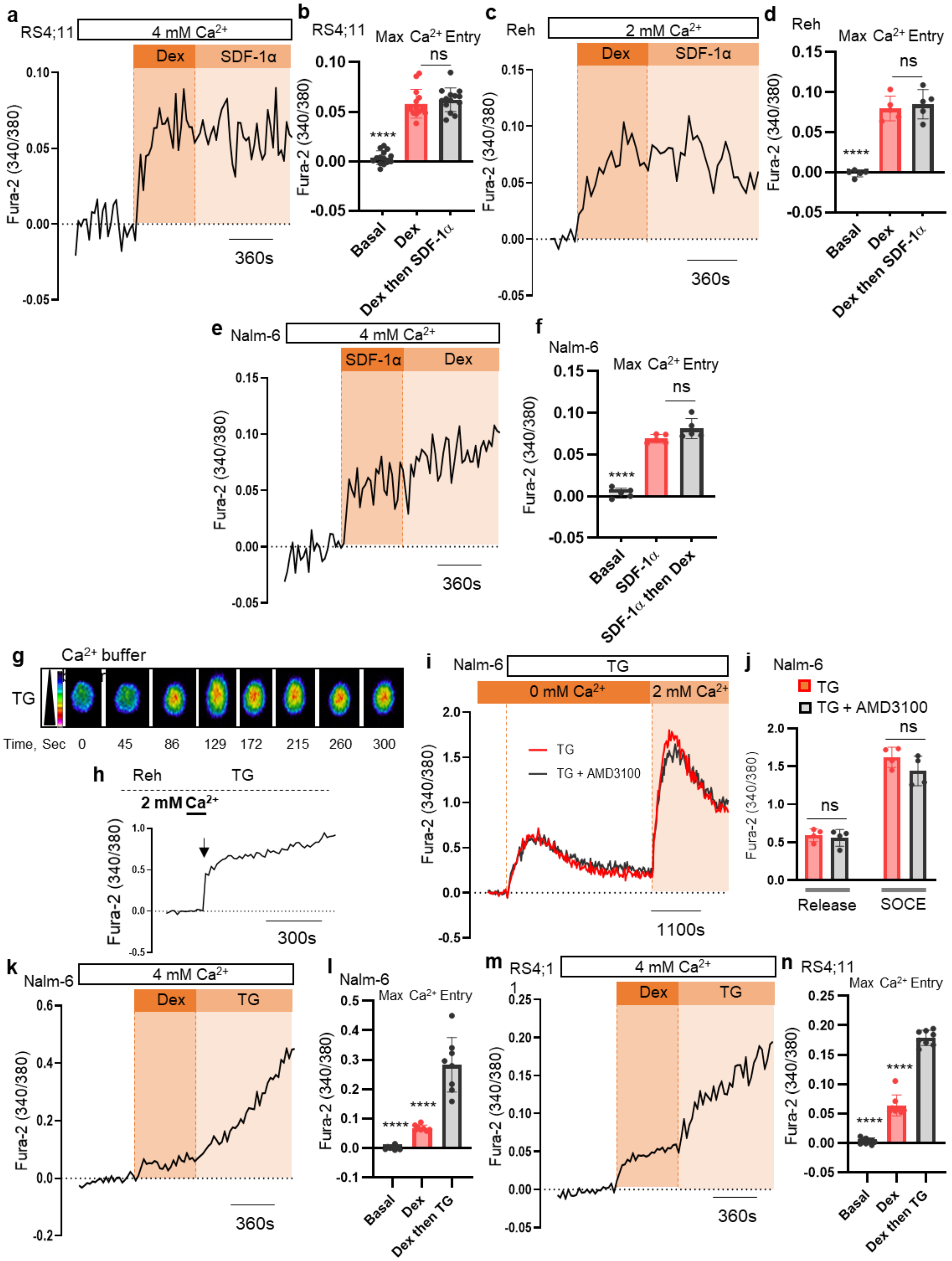

a RS4;11 4 mM Ca2+
Dex SDF-1α
Fura-2 (340/380)
0.10
0.05
0.00
-0.05
360s
b RS4;11 Max Ca2+ Entry
ns
****
Basal Dex Dex then SDF-1α
c Reh 2 mM Ca2+
Dex SDF-1α
0.15
360s
d Reh Max Ca2+ Entry
e Nalm-6 4 mM Ca2+
SDF-1α Dex
f Nalm-6 Max Ca2+ Entry
Basal SDF-1α SDF-1α then Dex
g Ca2+ buffer
TG
Time, Sec 0 45 86 129 172 215 260 300
h Reh TG
2 mM Ca2+
300s
i Nalm-6 TG
0 mM Ca2+ 2 mM Ca2+
TG
TG + AMD3100
1100s
j Nalm-6
TG
TG + AMD3100
Release SOCE
k Nalm-6 4 mM Ca2+
Dex TG
l Nalm-6 Max Ca2+ Entry
Basal Dex Dex then TG
m RS4;1 1 4 mM Ca2+
Dex TG
n RS4;11 Max Ca2+ Entry
Basal Dex Dex then TG

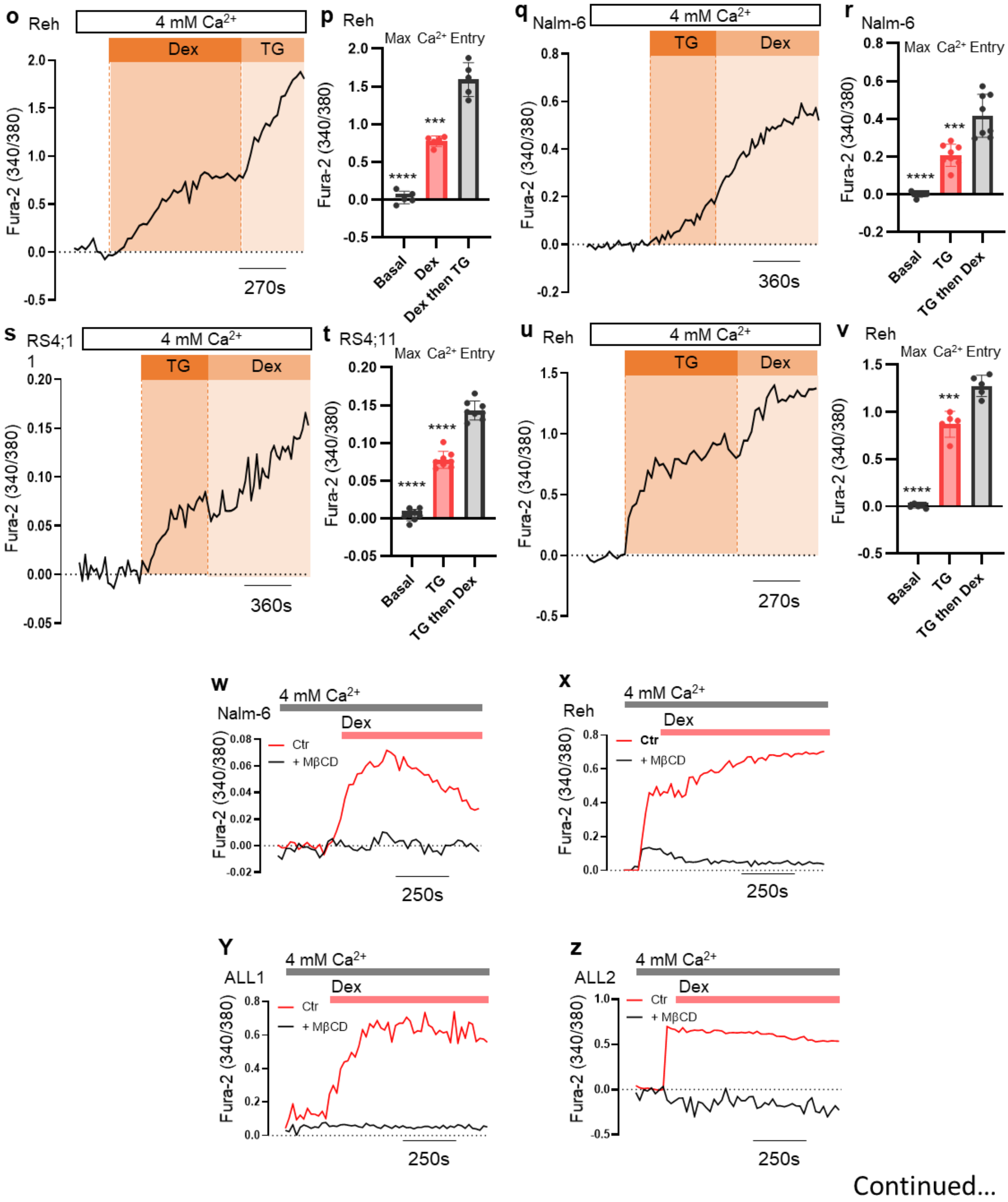

Continued...

Supplementary Figure 19. Dex-induced Ca^{2+} signaling in lipid rafts selectively through CXCR4 in B-ALL cells.

(**a**,**c**) Cytosolic Ca^{2+} measurements in RS4;11 (**a**) and Reh (**c**) cells stimulated with 125 nM Dex followed by addition of 100 nM SDF-1α in buffer containing Ca^{2+}. Data are represented as mean ± SEM. (**b**,**d**) Quantification of maximal Ca^{2+} entry from panel **a** and **c**, respectively. Data are mean ± SEM (n=14 for **b** and n=5 for **d**, independent experiments). (**e**) Cytosolic Ca^{2+} measurements in Nalm-6 cells stimulated with 100 nM SDF-1α followed by addition of 125 nM Dex in buffer containing 4 mM Ca^{2+}. Data are represented as mean ± SEM. (**f**) Quantification of maximal Ca^{2+} entry from panel (**e**). Data are mean ± SEM (n=5 independent experiments). (**g**) Colored time-lapse images of Reh cells show the changes in cytosolic Ca^{2+} evoked by 2 μM

thapsigargin (TG) in Ca^{2+}-containing buffer. (**h**) Cytosolic Ca^{2+} measurements in Reh cells stimulated with 2 μM TG in nominally Ca^{2+}-containing buffer (2 mM Ca^{2+}). (**i**) Cytosolic Ca^{2+} measurements in Nalm-6. Fura-2-loaded cells were stimulated by 2 μM TG in Ca^{2+}-free buffer followed by re-addition of extracellular Ca^{2+} (2 mM Ca^{2+} containing buffer). (**j**) Quantification of maximal Ca^{2+} release and SOCE from panel (**i**). Data are mean ± SEM (n=4 independent experiments). (**k**,**m**,**o**) Cytosolic Ca^{2+} measurements in Nalm-6 (**k**), RS4;11 (**m**) and Reh (**o**) cells stimulated with 125 nM Dex followed by addition of 2 μM TG in buffer containing 4 mM Ca^{2+}. Data are represented as mean ± SEM. (**l**,**n**,**p**) Quantification of maximal Ca2+ entry from panel (**k, m** and **o**, respectively). Data are mean ± SEM (n=8 for **l**,**n** and n=5 for **p**, independent experiments). (**q**,**s**,**u**) Cytosolic Ca^{2+} measurements in Nalm-6 (**q**), RS4;11 (**s**) and Reh (**u**) cells stimulated with 2 μM TG followed by addition of 125 nM Dex in buffer containing 4 mM Ca^{2+}. Data are represented as mean ± SEM. (**r**, **t**,**v**) Quantification of maximal Ca^{2+} entry from panel (**q, s** and **u**, respectively). Data are mean ± SEM (n=8 for **r**,**t** and n=5 for **v**, independent experiments). (**w-z**) Traces of cytosolic Ca^{2+} measurements in Nalm-6 cells (**w**), Reh cells (**x**), and primary B-ALL cells from two patients (**y**,**z**) stimulated with 125 nM Dex in Ca^{2+}-containing buffer (4 mM Ca^{2+}). Cells were preincubated with 10 μM methyl-β-cyclodextrin (MβCD) for 15 min before stimulation. Data are mean ± SEM (n=3 independent experiments). All statistical significance was calculated analyzed by two-tailed, unpaired Student's t test. ***p < 0.001 and ****p < 0.0001. ns, not significant. Source data are provided as a Source Data file.

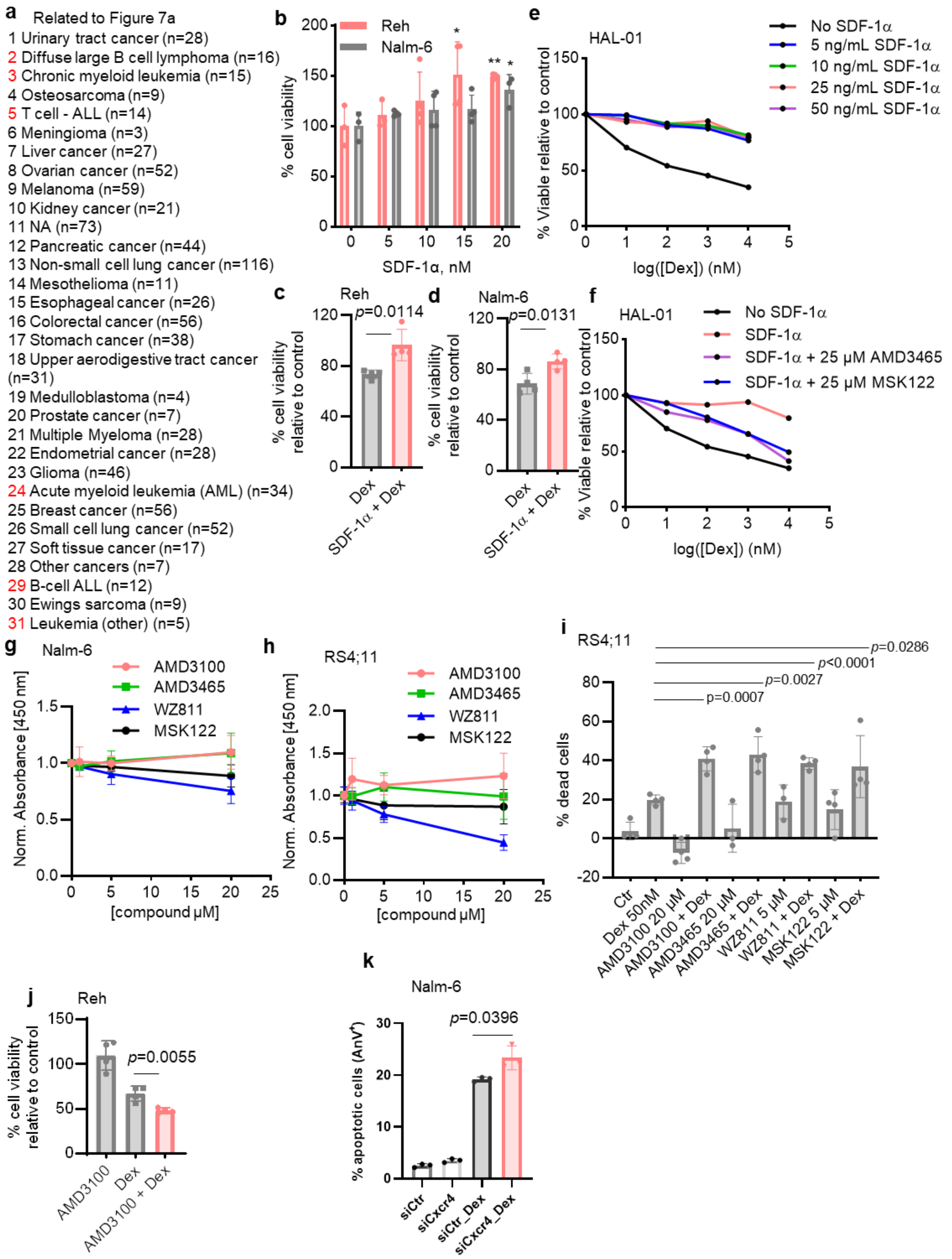

Supplementary Figure 20. CXCR4 signaling results in Dex resistance in B-ALL cell lines.

(**a**) Human cancer cell lines from the CCLE database ranked by Achilles CXCR4 shRNA score (high to low). Related to Figure 8a. Numbers are the same as those used in the graph in Figure 8a. Hematological malignancies are in red. 'n' represents the number of cell lines analyzed in each cancer type. (**b**) Viability normalized to the Ctr of Nalm-6 and Reh cells exposed for 48 hours with increasing concentrations of SDF-1α. Cell viability was determined by CCk-8 staining. Data are presented as means ± SEM (n=4 independent

experiments). Two-tailed, unpaired Student's t test and Mann-Whitney test are used. (**c**,**d**) Reh (**c**) and Nalm-6 (**d**) cells were exposed to 125 nM Dex for 48 hours in the presence or absence of 15 nM SDF-1α. Cell mortality was determined by CCk-8 staining. The values of control cells were considered as 100%. Two-tailed, unpaired Student's t test. Data are presented as means ± SEM (n=4 independent experiments). (**e**) Viability relative to Ctr of HAL-01 cells exposed for 48 hours to Dex in the presence or absence of increasing concentrations of SDF-1α. Data are presented as means ± SEM (n=3 independent experiments). (**f**) Viability of HAL-01 cells exposed to Dex in the presence of 25 ng/mL SDF-1α in the presence or absence of CXCR4 antagonists. The "No SDF-1α" (black line) and the 25 ng/mL SDF-1α (red line) conditions were replotted from panel (**e**). Data are presented as means ± SEM (n=3 independent experiments). (**g**,**h**) Viability normalized to the Ctr of Nalm-6 cells (**g**) and RS4;11 cells (**h**) exposed for 48 hours with increasing concentrations of CXCR4 antagonists. Data are presented as means ± SEM (n=4 independent experiments). (**i**) RS4;11 cells treated with Dex, AMD3100, AMD3465, WZ811, and MSK122 alone or in combination. Cell mortality was determined by CCk-8 staining after 48 hours of treatment. The percentage of dead cells was established after normalizing cells on Ctr cells. Two-tailed, unpaired Student's t test. Data are presented as means ± SEM (n=4 independent experiments). (**j**) Reh cells were exposed to125 nM Dex for 48 hours in the presence or absence of 25 µM AMD3100. Cell mortality was determined by CCk-8 staining. The values of control cells were considered as 100%. Two-tailed, unpaired Student's t test; n=4 independent experiments. Data are presented as means ± SEM. (**k**) Annexin V-positive cells were measured by flow cytometry after 2 days of 125 nM Dex treatment in Nalm-6 cells transfected with either siCtr or siCXCR4. Data are presented as means ± SEM (n=3 independent experiments). Statistical significance was analyzed by two-tailed, unpaired Student's *t* test. *p < 0.05, **p < 0.01, ***p < 0.001 and ****p < 0.0001. Source data are provided as a Source Data file.

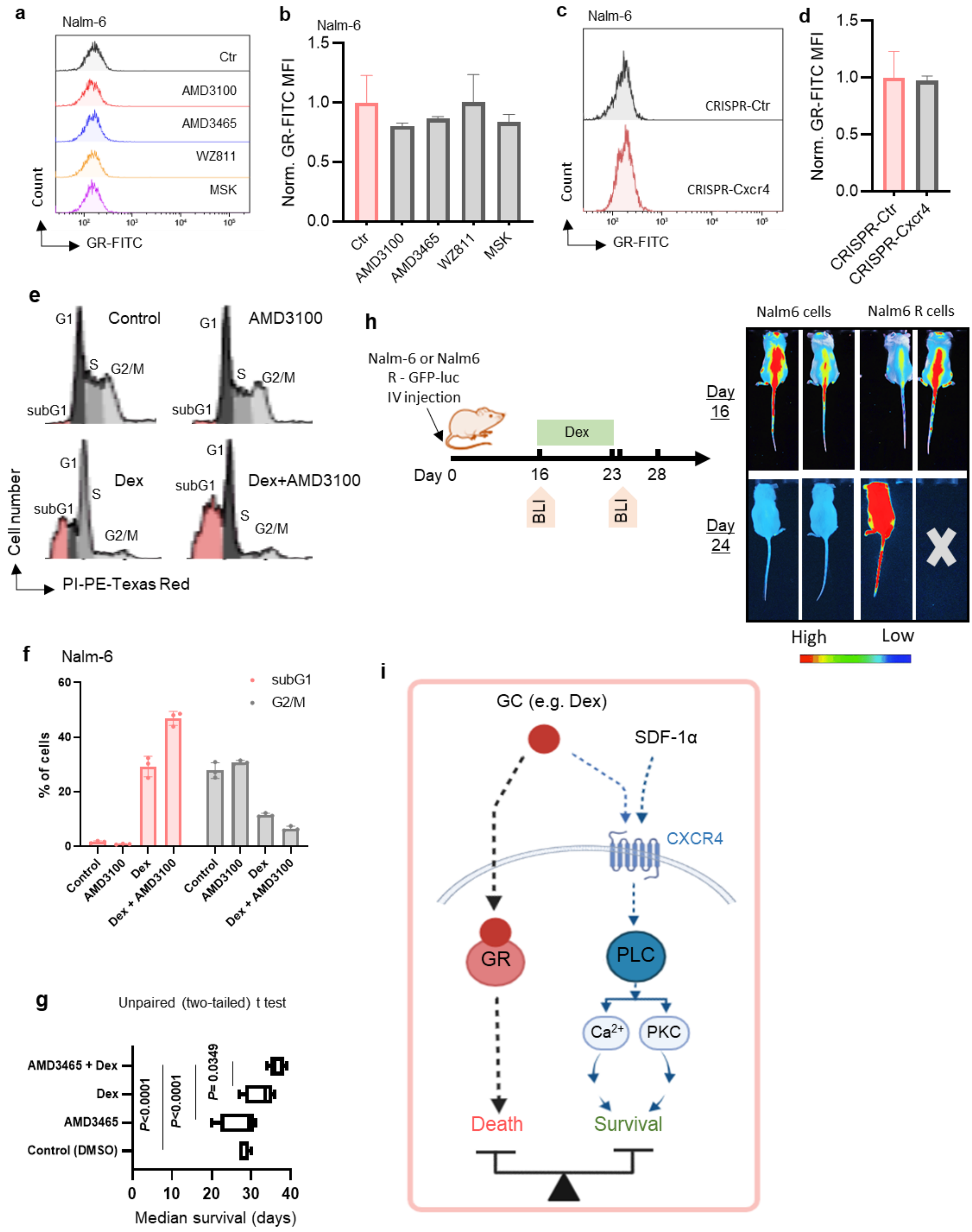

Supplementary Figure 21. CXCR4 Inhibition enhances Dex sensitivity in B-ALL cell lines.

(**a**,**b**) Representative flow cytometry plots (**a**) and normalized quantification (**b**) of GR MFI stimulated with CXCR4 antagonists or Ctr for 24 hours in Nalm-6 cells. Data are mean ± SEM (**b**: n = 4 for Ctr and n=2 for antagonist; n-values correspond to independent experiments). (**c**,**d**) Representative flow cytometry plots (**c**) and normalized quantification (**d**) of GR MFI stimulated with CXCR4 antagonists or Ctr for 24 hours in Nalm-6 cells transduced with either CRISPR-Ctr or CRISPR-Cxcr4. Data are mean ± SEM (n=2 independent

experiments for **d**). (**e**) Cell cycle analysis of Nalm-6 cells before and after stimulation for 72 hours with 125 nM Dex or AMD3100 alone or combination. Representative flow cytometry plots of different cell cycle phases. (**f**) Quantification of subG1 and G2/M cell cycle phases from panel **e**. Statistical significance was analyzed by two-tailed, unpaired Student's t test. Data are mean ± SEM (n=3 technical replicate). The "Ctr" and the "Dex" conditions were replotted from Figure 5**i**. (**g**) Average of the median survival over time is shown for the different treatment groups after 2 weeks of treatment as in Figure 8c. Data are the mean ± SEM from (n = 4 for Ctr, AMD3465 and n=6 for Dex and Dex+AMD; n-values correspond to individual mice). Statistical significance was analyzed by two-tailed, unpaired Student's t test. Boxplots show the mean, median, and the 75th to 25th percentiles. (**h**) In vivo validation of our Nalm-6 resistant model. Experimental design of NSG mice treatment experiments and an example of bioluminescence imaging (BLI) of tumor challenge in Nalm-6 GFP/luc cells and Nalm-6 R cells. (**i**) Proposed model for the mechanism by which GC induce their own resistance in B-ALL cells. Dex treatment leads to GR translocation, which induces a transcriptional program resulting in lymphoid cell death (therapeutic effect). Along with this pathway, Dex also provokes internalization of CXCR4 and subsequent triggering of a pro-survival activity (paradoxical effect) driven by the PLC signalosome. This latter pathway may blunt the anticancer efficacy of chemotherapy (Created with BioRender.com). Source data are provided as a Source Data file.

Supplementary Table 1

Clinical and biological characteristics of Patients with ALL

**Clinical characteristics**	**Patients** (n=44)
Age at diagnosis (months)	80.64 ± 55.65
Peripheral blood % of blasts	86.88 ± 11.26
Bone marrow % of blasts	85.68 ± 10.30
Immunological phenotype (B)	22
Immunological phenotype (T)	22
Karyotype abnormalities	normal
Glucocorticoids (Sensitive)	17
Glucocorticoids (resistant)	27
Chemotherapy (Sensitive)	22
Chemotherapy (resistant)	22
MRD at day 35 (Negative)	12
MRD at day 35 (Positive)	18
Relapse	60%

MRD: minimal residual disease

Supplementary Table 2

REAGENT or RESOURCE	SOURCE	IDENTIFIER
Chemicals, Materials		
Poly-D-lysine	Sigma-Aldrich	P6407-5MG
2-aminoethoxydiphenyl borate (2-APB)	Sigma-Aldrich	D9754-1G
2-aminoethoxydiphenyl borate (2-APB)	Abcam	ab120124
Ethylene glycol tetraacetic acid, EGTA	Sigma-Aldrich	E8145-10G
U73122	Abcam	ab120998
U73122	Enzo Life Sciences	BML-ST391-0005
U73122	Tocris	1268/10
U73122	Selleckchem	S8011
Manoalide	Enzo Life Sciences	BML-EI177-0001
3-Nitrocoumarin	Medchemexpress	HY 111919
SPK-601	Medchemexpress	HY-70083
D609	Medchemexpress	HY 70072
Edelfosine	Enzo Life Sciences	BML-L108-0005
Cyclosporin A (CsA)	Medchemexpress	HY-B0579
Tacrolimus / FK506	Medchemexpress	HY-13756
MCD (β-methylcyclodextrin)	Sigma-Aldrich	C4555-1G
MSX-122	Selleckchem	S6617
AMD3100	Abcam	ab120718
AMD3100	Selleckchem	S8030
AMD3465 hexahydrobromide	Selleckchem	S2879
AMD3465 hexahydrobromide	Medchemexpress	HY-15971
AMD3100	Medchemexpress	HY-50912
WZ811	Selleckchem	S2912
Sotrastaurin	Selleckchem	S2791
Go6983	Selleckchem	S2911
Go6976	Selleckchem	S7119
GF109203X	Selleckchem	S7208
GF109203X	Abcam	ab144264
Mifepristone (RU486)	Abcam	ab120356

Ionomycin (free acid)	Abcam	ab120370
Thapsigargin	Enzo Life Sciences	BML-PE180-0001
Thapsigargin	Abcam	ab120286
Fura-2/AM	Abcam	ab120873
Fura Red™, AM	Fisher (Life Tech)	F3021
Fura-2 QBT Calcium Kit (Bulk)	Molecular Devices	R8198
Fluo-4 AM	Abcam	ab241082
YM-58483 (BTP2)	Abcam	ab144413
CM-4620 (Zegocractin)	Medchemexpress	HY-101942
GSK-7975A	GLIXX LABS	GLXC-03243
Synta66	GLIXX LABS	GLXC-03244
Dexamethasone	Enzo Life Sciences	BML-EI126-0001
Ac-DEVD-AFC, Caspase-3 substrate	Enzo Life Sciences	ALX-260-032-M005
Human SDF-1 alpha (CXCL12)	Peprotech	300-28A
96 Well Black/Clear Bottom Plate	Grosseron	CG3603
96 Well, Flex Pipet Tips (Black)	Molecular Devices	9000-0911
Recombinant Human CXCL12/SDF-1a	R&D Systems	350-NS-010/CF
24-well Black, Krystal glass bottom	Proteigene	324041
CHAPS	Abcam	ab141396
Cell Counting Kit - 8	Sigma-Aldrich	96992-500
Cell Counting Kit 8	Abcam	ab228554
Cell Counting Kit 8	Medchemexpress	HY-K0301
Adenosine 5'-triphosphate (sodium sait)	Bertin Technologies	WP-14498
Cell-Tak CellTissue	Corning B.V.	354240
Cell-Tak CellTissue	Fisher Scientific	10317081
XenoLight D-Luciferin-K+Salt BL Substrate	Elmer Perkin	122799
True-Phos Perm Buffer	Ozyme	BLE425401
Fixation Buffer	Ozyme	BLE420801
Critical Commercial Assays		
Annexin V-FITC Apoptosis Detection Kit	Abcam	ab14085
PKC Kinase Activity Assay Kit	Abcam	ab139437
Dynabeads Untouched Human B Cells Kit	Invitrogen	11351D
FlowCellect Cytochrome C Kit	Millipore	FCCH100110
EnzChek™ Direct PLC Assay Kit	Invitrogen	E10215

CellTrace Violet Cell Proliferation Kit	Invitrogen	C34557
PKC Kinase activity kit	Enzo Life Sciences	ADI-EKS-420A
MycoAlert PLUS detection kit	Lonza	LT07-705
Sequencing reagents		
Multiplex Oligos for Illumina®	New England Biolabs	E7335S
Poly(A) mRNA Magnetic Isolation	New England Biolabs	E7490S
Ultra™ II Directional RNA Library Prep	New England Biolabs	E7760S
Multiplex Oligos for Illumina®	New England Biolabs	E7335S
NextSeq 500/550 High Output	Illumina	20024907
Carbamoylcholine chloride	Tocris	2810/100
Puromycin dihydrochloride 31	Santa Cruz	sc-108071
Blasticidin S HCl solution 37	Santa Cruz	sc-495389
Hygromycin B solution 97	Santa Cruz	sc-29067
Experimental Models: Cell Lines, Organisms/Strains		
Reh cells	DSMZ	ACC 22
Nalm-6 cells	DSMZ	ACC 128
RS4;11 cells	DSMZ	ACC 508
Hal-01	DSMZ	ACC 610
RCH-ACV	DSMZ	ACC 548
Nalm-6 (transduced)	ATCC	CRL-3273
Nalm-6 S	This paper	N/A
Nalm-6 R	This paper	N/A
Nalm-6 : CRISPR-Ctr cells	This paper	N/A
Nalm-6 : CRISPR-Cxcr4 cells	This paper	N/A
RS4;11 : CRISPR-Ctr cells	This paper	N/A
RS4;11 : CRISPR-Cxcr4 cells	This paper	N/A
NOD scid gamma	Charles River	JAX® Mice Strain
NOD scid gamma	University pet store	U1234
Antibodies		
GR (G-5) FITC	Santa Cruz	sc-393232
CD184 (CXCR4) Antibody (12G5), APC	Ebioscience	17-9999-42
APC anti-human CD184 (CXCR4)	Biolegend	306510
CD184 (CXCR4) Antibody (12G5), APC	Invitrogen	17-9999-42
APC Mouse IgG1, kappa Isotype control	Biolegend	400119

APC Mouse IgG1, kappa Isotype control	BD Biosciences	554681
PE Mouse IgG1, kappa Isotype control	Biolegend	400111
APC Mouse Anti-Human CD184 (CXCR4)	BD Biosciences	BDB555976
PLC γ2 (B-10) Alexa Fluor® 647	Santa Cruz	sc-5283
PE Mouse Anti-PLCγ2 (pY759)	BD Biosciences	BD558490
APC Phospho-PLCγ2 (Tyr759)	ThermoFisher	17-9866-42
PLC γ1 (E-12) FITC	Santa Cruz	sc-7290 FITC
Anti-Mouse CD45	BD Biosciences	561018
Human CD19-Pacific Blue	Beckman Coulter	B49213
Transfection, Plasmids, Buffer		
ON-TARGETplus Human CXCR4 siRNA	Dharmacon	J-005139-08-0002
ON-TARGETplus Non-targeting Pool	Dharmacon	D-001810-10-05
DharmaFECT 1 Transfection Reagent	Dharmacon	T-2001-02
CXCR-4 CRISPR/Cas9 KO Plasmid (h)	Santa Cruz	sc-400254
Control CRISPR/Cas9 Plasmid	Santa Cruz	sc-418922
UltraCruz Transfection Reagent	Santa Cruz	sc-395739
PLC γ1 siRNA (h)	Santa Cruz	sc-29452
PLCγ2 siRNA (h)	Santa Cruz	sc-36268
Control siRNA-A	Santa Cruz	sc-37007
siRNA Transfection Reagent	Santa Cruz	sc-29528
Software		
GraphPad Prism 8 Software	Graph Pad	N/A
SoftMax® Pro 7.1 Software	Molecular Devices	N/A
Kaluza Software	Beckman Coulter	N/A
BD FACSDiva™ Software	BD Biosciences	N/A
