# Supplementary material for: Glucocorticoids paradoxically promote steroid resistance in B cell acute lymphoblastic leukemia through CXCR4/PLC signaling
Source: Nat Commun. 2024 May 29;15:4557. doi: 10.1038/s41467-024-48818-9 (PMC11136999; doi:10.1038/s41467-024-48818-9)
Supplement: Supplementary file 2 — Reporting Summary [file 41467_2024_48818_MOESM2_ESM.pdf]

nature portfolio

Corresponding author(s): Souleymane Abdoul-Azize

Last updated by author(s): Apr 4, 2024

Reporting Summary

Nature Portfolio wishes to improve the reproducibility of the work that we publish. This form provides structure for consistency and transparency in reporting. For further information on Nature Portfolio policies, see our Editorial Policies and the Editorial Policy Checklist.

Statistics

For all statistical analyses, confirm that the following items are present in the figure legend, table legend, main text, or Methods section.

n/a	Confirmed	
[ ]	[x]	The exact sample size (*n*) for each experimental group/condition, given as a discrete number and unit of measurement
[ ]	[x]	A statement on whether measurements were taken from distinct samples or whether the same sample was measured repeatedly
[ ]	[x]	The statistical test(s) used AND whether they are one- or two-sided<br>*Only common tests should be described solely by name; describe more complex techniques in the Methods section.*
[x]	[ ]	A description of all covariates tested
[ ]	[x]	A description of any assumptions or corrections, such as tests of normality and adjustment for multiple comparisons
[ ]	[x]	A full description of the statistical parameters including central tendency (e.g. means) or other basic estimates (e.g. regression coefficient) AND variation (e.g. standard deviation) or associated estimates of uncertainty (e.g. confidence intervals)
[ ]	[x]	For null hypothesis testing, the test statistic (e.g. *F*, *t*, *r*) with confidence intervals, effect sizes, degrees of freedom and *P* value noted<br>*Give P values as exact values whenever suitable.*
[x]	[ ]	For Bayesian analysis, information on the choice of priors and Markov chain Monte Carlo settings
[x]	[ ]	For hierarchical and complex designs, identification of the appropriate level for tests and full reporting of outcomes
[ ]	[x]	Estimates of effect sizes (e.g. Cohen's *d*, Pearson's *r*), indicating how they were calculated

Our web collection on statistics for biologists contains articles on many of the points above.

Software and code

Policy information about availability of computer code

Data collection	Bioluminescent imaging data was acquired using Vilber Smart In Vivo Imaging System (Vilber lourmat).<br>Calcium time-lapse imaging data was acquired using Leica DM16OOO B inverted microscope (Leica) equipped with SENSICAM EM camera.<br>Cell viability, Calcium, and Caspase-3 Activity measurements were acquired using SoftMax Pro 7.1 software in the Flexstation3 multiplate reader (Molecular Devices).<br>Flow cytometry data was acquired using FACSDiva in the BD LSR Fortessa Flow Cytometer (BD Bioscience) and FACSAria cell sorter (BD Biosciences) for cell sorting and BD FACSCanto II Flow Cytometer (BD Biosciences) for apoptosis analysis.<br>RNA quality and quantity was analyzed on a Bioanalyzer 2100 (Agilent Technologies) and the Qubit 3.0 device (Thermo Scientific). RNA-Seq libraries were prepared using NEBNext Ultra II Directional RNA Library Kit for Illumina (New England Biolabs). RNA-seq libraries were pooled equimolarly, and loaded on Illumina NextSeq500/550 using 2*75bp sequencing to generate 30M read pairs on average per sample.<br>Fluorescence intensity and absorbance data were collected using SAFAS Xenius XC Spectrofluorometer (MC 98000 Monaco) or Flexstation3 multiplate reader (Molecular Devices).
Data analysis	For RNAseq gene expression analysis, bioinformatics analysis was carried out using nf-core/rnaseq v3.1 analysis pipeline (https://nf-co.re/rnaseq) (Ewels et al.,Nat Biotechnol 2020:38(3):276-278) to generate multi quality control report that uses the STAR v2.6.1d and SALMON v1.4.0 tools for alignment. Differential expression analysis, based on a model using the binomial negative distribution, was performed with DESeq2 tool to evaluate significant counts in cells treated with U73122 compared to untreated (control) cells.<br>Differences in gene expression were considered significant if padj<0.05. Gene expression signatures and canonical pathway analyses were performed using GO, KEGG, Reactome, Biocarta and Wiki's pathways analysis and gene set enrichment analysis (GSEA, https://software.broadinstitute.org/gsea/index.jsp).

Mice image processing and quantification were performed using Newton 7.0 Software (Vilber smart Imaging).
Flow cytometry data was analyzed using the Kaluza software (kaluza A85810 AB).
Venn diagram depicting was carried out using online tool (https://bioinformatics.psb.ugent.be/webtools/Venn/).
All data was visualized with Graphpad Prism and statistical analysis was done using the Prism Software (Graphpad) v8.0.2.

For manuscripts utilizing custom algorithms or software that are central to the research but not yet described in published literature, software must be made available to editors and reviewers. We strongly encourage code deposition in a community repository (e.g. GitHub). See the Nature Portfolio guidelines for submitting code & software for further information.

Data

Policy information about availability of data

All manuscripts must include a data availability statement. This statement should provide the following information, where applicable:

- Accession codes, unique identifiers, or web links for publicly available datasets
- A description of any restrictions on data availability
- For clinical datasets or third party data, please ensure that the statement adheres to our policy

RNA-Seq data generated for this study have been deposited in the GEO database under
accession number GSE214990 [https://www-ncbi-nlm-nih-gov.proxy.insermbiblio.inist.fr/geo/query/acc.cgi?acc=GSE214990]
Additional available RNA-Seq data sets used in this study were downloaded from GEO or ArrayExpress using the following accession numbers:
GSE655 [https://www-ncbi-nlm-nih-gov.proxy.insermbiblio.inist.fr/geo/query/acc.cgi?acc=GSE655]
GSE656 [https://www-ncbi-nlm-nih-gov.proxy.insermbiblio.inist.fr/geo/query/acc.cgi?acc=GSE656]
GSE5820 [https://www-ncbi-nlm-nih-gov.proxy.insermbiblio.inist.fr/geo/query/acc.cgi?acc=GSE5820]
GSE28460 [https://www-ncbi-nlm-nih-gov.proxy.insermbiblio.inist.fr/geo/query/acc.cgi?acc=GSE28460]
GSE18497 [https://www-ncbi-nlm-nih-gov.proxy.insermbiblio.inist.fr/geo/query/acc.cgi?acc=GSE18497]
E-MTAB-7781 [https://www.ebi.ac.uk/biostudies/arrayexpress/studies/E-MTAB-7781?query=E-MTAB-7781]
GSE13159 [https://www-ncbi-nlm-nih-gov.proxy.insermbiblio.inist.fr/geo/query/acc.cgi?acc=GSE13159]
GSE63157 [https://www-ncbi-nlm-nih-gov.proxy.insermbiblio.inist.fr/geo/query/acc.cgi?acc=GSE63157]
GSE45547 [https://www-ncbi-nlm-nih-gov.proxy.insermbiblio.inist.fr/geo/query/acc.cgi?acc=GSE45547]
GSE37642 [https://www-ncbi-nlm-nih-gov.proxy.insermbiblio.inist.fr/geo/query/acc.cgi?acc=GSE37642]
GSE10846 [https://www-ncbi-nlm-nih-gov.proxy.insermbiblio.inist.fr/geo/query/acc.cgi?acc=GSE10846]
GSE13204 [https://www-ncbi-nlm-nih-gov.proxy.insermbiblio.inist.fr/geo/query/acc.cgi?acc=GSE13204]
GSE7186 [https://www-ncbi-nlm-nih-gov.proxy.insermbiblio.inist.fr/geo/query/acc.cgi?acc=GSE7186]
survival data from the R2 database (http://r2.amc.nl)
Additionally, published gene expressions analyzed in this study were
downloaded from Cancer Cell Line Encyclopedia (CCLE, https://sites.broadinstitute.org/ccle/), and Gene Expression Profiling Interactive Analysis (GEPIA, http://gepia.cancer-pku.cn/index.html).

Research involving human participants, their data, or biological material

Policy information about studies with human participants or human data. See also policy information about sex, gender (identity/presentation), and sexual orientation and race, ethnicity and racism.

Reporting on sex and gender	No overt gender differences were observed in all experiments.
Reporting on race, ethnicity, or other socially relevant groupings	The samples in this study were all obtained from children aged 80.64 ± 55.65 (months) at the time of diagnosis.
Population characteristics	Population characteristics are given in SupplementaryTable 1
Recruitment	Retrospective study based on the “Research” biological collection of the pediatric immuno-hemato-oncology department. Samples of primary ALL cells are obtained during traditional assessments as part of the department's authorized biocollection maintained at the Institute of Clinical Biology, Medical University of Rouen, France.<br>Informed written consent was obtained from patients or their guardians at the time of sample collection.
Ethics oversight	Protocols was approved by the institutional review board of Rouen University and Hospital Center, in accordance with Declaration of Helsinki principles.

Note that full information on the approval of the study protocol must also be provided in the manuscript.

Field-specific reporting

Please select the one below that is the best fit for your research. If you are not sure, read the appropriate sections before making your selection.

[x] Life sciences [ ] Behavioural & social sciences [ ] Ecological, evolutionary & environmental sciences

For a reference copy of the document with all sections, see nature.com/documents/nr-reporting-summary-flat.pdf

Life sciences study design

All studies must disclose on these points even when the disclosure is negative.

Sample size	No statistical methods were used to predetermine sample sizes, but our fundamental findings were confirmed with a variety of different methods to avoid any method-specific bias. In addition, each figure legend contains the information how many samples were used for the described experiment. Our sample sizes are similar to those reported in previous publications (Abdoul-azize, Set al. 2018; Serafin, Vet al. 2017; Johnson, M. et al. 2020)
Data exclusions	No data exclusion was performed
Replication	All experiments were done at least in two independent experiments but generally more than 2 with similar results. Numbers of mice was between 4-6 per condition.
Randomization	Animals was assigned randomly to experimental conditions with similar BLI level. For experiments other than in vivo experiments, randomization was not relevant to this study since samples did not need to be allocated to specific groups.
Blinding	For in vivo experiments, the analysis was performed blindly. For experiments other than in vivo experiments, blinding was not relevant because there was no group allocation during data collection and analysis.

Reporting for specific materials, systems and methods

We require information from authors about some types of materials, experimental systems and methods used in many studies. Here, indicate whether each material, system or method listed is relevant to your study. If you are not sure if a list item applies to your research, read the appropriate section before selecting a response.

Materials & experimental systems

n/a	Involved in the study	
☐	☒	Antibodies
☐	☒	Eukaryotic cell lines
☒	☐	Palaeontology and archaeology
☐	☒	Animals and other organisms
☒	☐	Clinical data
☒	☐	Dual use research of concern
☒	☐	Plants

Methods

n/a	Involved in the study	
☒	☐	ChIP-seq
☐	☒	Flow cytometry
☒	☐	MRI-based neuroimaging

Antibodies

Antibodies used

GR (G-5) FITC, Santa Cruz, sc-393232, clone G-5
CD184 (CXCR4) Antibody (12G5), APC Ebioscience, 17-9999-42, clone 12G5
APC anti-human CD184 (CXCR4), Biolegend, 306510, clone 12G5
APC Mouse IgG1, kappa Isotype control, Biolegend, 400119, clone MOPC-21
APC Mouse IgG1, kappa Isotype control, BD Biosciences, 554681, clone MOPC-21
PE Mouse IgG1, kappa Isotype control, Biolegend, 400111, clone MOPC-21
APC Mouse Anti-Human CD184 (CXCR4), BD Biosciences, BDB555976, clone 12G5
PLC y2 (B-10) Alexa Fluor® 647, Santa Cruz sc-5283, clone B-10
PE Mouse Anti-PLCy2 (pY759), BD Biosciences, BD558490, clone K86-689.37
APC Phospho-PLCy2 (Tyr759), ThermoFisher, 17-9866-42, clone 4NPRN4
Anti-Cytochrome c-FITC antibody (FlowCellect Cytochrome C Kit) Millipore FCCH100110
PLC yl (E-12) FITC Santa Cruz sc-7290 FITC, clone E-12
Anti Mouse CD45, BD Biosciences 561018, clone 30-F11
Human CD19 Pacific Blue, Beckman Coulter B49213, clone J3-119

Validation

Validation statements and other antibody information:
GR (G-5) FITC, Santa Cruz, sc-393232, clone G-5, https://www.scbt.com/fr/p/gr-antibody-g-5
CD184 (CXCR4) Antibody (12G5), APC Ebioscience, 17-9999-42, clone 12G5, https://www.thermofisher.com/antibody/product/CD184-CXCR4-Antibody-clone-12G5-Monoclonal/17-9999-42
APC anti-human CD184 (CXCR4), Biolegend, 306510, clone 12G5, https://www.biolegend.com/fr-ch/clone-search/apc-anti-human-cd184-cxcr4-antibody-539?GroupID=BLG8071
APC Mouse IgG1, kappa Isotype control, Biolegend, 400119, clone MOPC-21, https://www.biolegend.com/fr-lu/clone-search/apc-mouse-igg1-kappa-isotype-ctrl-1404
APC Mouse IgG1, kappa Isotype control, BD Biosciences, 554681, clone MOPC-21, https://www.bdbiosciences.com/en-us/products/reagents/flow-cytometry-reagents/research-reagents/flow-cytometry-controls-and-lysates/apc-mouse-igg1-isotype-control.554681
PE Mouse IgG1, kappa Isotype control, Biolegend, 400111, clone MOPC-21, https://www.biolegend.com/nl-be/products/pe-mouse-

igg1-kappa-isotype-ctrl-1408
APC Mouse Anti-Human CD184 (CXCR4), BD Biosciences, BDB555976, clone 12G5, https://www.bdbiosciences.com/en-us/products/reagents/flow-cytometry-reagents/research-reagents/single-color-antibodies-ruo/apc-mouse-anti-human-cd184.555976
PLC y2 (B-10) Alexa Fluor® 647, Santa Cruz sc-5283, clone B-10, https://www.scbt.com/fr/p/plc-gamma2-antibody-b-10
PE Mouse Anti-PLCy2 (pY759), BD Biosciences, BD558490, clone K86-689.37, https://www.bdbiosciences.com/en-us/products/reagents/flow-cytometry-reagents/research-reagents/single-color-antibodies-ruo/pe-mouse-anti-plc-2-py759.558490
APC Phospho-PLCy2 (Tyr759), ThermoFisher, 17-9866-42, clone 4NPRN4, https://www.thermofisher.com/antibody/product/Phospho-PLCG2-Tyr759-Antibody-clone-4NPRN4-Monoclonal/17-9866-42
Anti-Cytochrome c-FITC antibody (FlowCellect Cytochrome C Kit) Millipore FCCH100110, https://www.merckmillipore.com/FR/fr/search/FCCH100110?search=&TrackingSearchType=SB+-+Search+Box&SearchContextPageletUUID=&SearchTerm=FCCH100110&search=
PLC yl (E-12) FITC Santa Cruz sc-7290 FITC, clone E-12, https://www.scbt.com/fr/p/plc-gamma1-antibody-e-12
Anti Mouse CD45, BD Biosciences 561018, clone 30-F11, https://www.bdbiosciences.com/en-us/products/reagents/flow-cytometry-reagents/research-reagents/single-color-antibodies-ruo/apc-rat-anti-mouse-cd45.561018
Human CD19 Pacific Blue, Beckman Coulter B49213, clone J3-119, https://www.beckman.fr/reagents/coulter-flow-cytometry/antibodies-and-kits/single-color-antibodies/cd19/B49213

Eukaryotic cell lines

Policy information about cell lines and Sex and Gender in Research

Cell line source(s)	Reh, Nalm-6, RS4;11, HAL-01 and RCH-ACV cell lines from DSMZ, Nalm-6 (transduced) from ATCC. Key ressources table is provided with this paper
Authentication	All cell lines were authenticated via short tandem repeat (STR) methodology.<br>Nalm-6-BLIV cells were evaluated by luciferase and GFP expression. Successful CRISPR cell transfections were confirmed by GFP detection and sorted step to eliminate GFP-negative cells.
Mycoplasma contamination	All cell lines were routinely tested for mycoplasma contamination using the MycoAlert PLUS detection kit (Lonza, LT07-705). All cell lines were negative for mycoplasma in our tests.
Commonly misidentified lines (See ICLAC register)	No commonly misidentified cell lines were used.

Animals and other research organisms

Policy information about studies involving animals; ARRIVE guidelines recommended for reporting animal research, and Sex and Gender in Research

Laboratory animals	Animal experiments were conducted following protocols that were approved by the regional ethics committee for animal experimentation (CENOMEXA, APAFIS#27919). NSG mice (Charles River) were used at 6 to 12 weeks old and were age-matched. NSG mice injected intravenously with Nalm-6 GFP/luciferase cells were treated after engraftment was established (luminescent flux assessed by bioluminescence imaging). Mice were randomized into four groups to equally distribute the leukemic burden (as assessed by bioluminescence) and weight in the different treatment groups. . AMD3465 (dissolved in PBS) was administered subcutaneously (100 µL per mouse) at a concentration of 5 mg/kg for 3 days. U73122 (dissolved in DMSO then in PBS) was administered intraperitoneally (100 µL per mouse) at a concentration of 15 mg/kg for 3 days. The mice in the control and dexamethasone (Dex) only groups were treated with solvent as second treatment. Dex (Enzo Life Sciences, catalog no. BML-El126) was administered by intraperitoneal injection at a dose of 15 mg/kg/day in a cycle of 4-5 days on/3 days off during the 2 weeks of treatment. The endpoint for leukemic-free survival was reached when mice had their hind limbs paralyzed.
Wild animals	No wild animals were used.
Reporting on sex	Experiments were performed in male and female mice. Animals are treated as the same in this study. No overt gender differences were seen in all experiments.
Field-collected samples	No field collected samples were used for this study.
Ethics oversight	Regional ethics committee for animal experimentation (CENOMEXA)

Note that full information on the approval of the study protocol must also be provided in the manuscript.

Plants

Seed stocks

Report on the source of all seed stocks or other plant material used. If applicable, state the seed stock centre and catalogue number. If plant specimens were collected from the field, describe the collection location, date and sampling procedures.

Novel plant genotypes

Describe the methods by which all novel plant genotypes were produced. This includes those generated by transgenic approaches, gene editing, chemical/radiation-based mutagenesis and hybridization. For transgenic lines, describe the transformation method, the number of independent lines analyzed and the generation upon which experiments were performed. For gene-edited lines, describe the editor used, the endogenous sequence targeted for editing, the targeting guide RNA sequence (if applicable) and how the editor was applied.

Authentication

Describe any authentication procedures for each seed stock used or novel genotype generated. Describe any experiments used to assess the effect of a mutation and, where applicable, how potential secondary effects (e.g. second site T-DNA insertions, mosiacism, off-target gene editing) were examined.

Flow Cytometry

Plots

Confirm that:

- [x] The axis labels state the marker and fluorochrome used (e.g. CD4-FITC).
- [x] The axis scales are clearly visible. Include numbers along axes only for bottom left plot of group (a 'group' is an analysis of identical markers).
- [x] All plots are contour plots with outliers or pseudocolor plots.
- [x] A numerical value for number of cells or percentage (with statistics) is provided.

Methodology

Sample preparation

Normal B cell were isolated by Ficoll-Paque Plus (GE Healthcare) and Dynabeads Untouched Human B cells kit (Life Technologies).
Staining of cell surface or intracellular antigens with fluorescently labeled antibodies was carried out on ice or RT.
For Phospho flow cytometry, ALL cells were treated with or without CXCR4 antagonists for 30 minutes, and then stimulated with Ctr or 125 nM Dex for 5 minutes.
ALL cell lines were also treated with or without test compounds for 24 h for others experiements (eg. Cytochrome c release).

Instrument

Samples were acquired on BD LSR Fortessa or BD FACSCanto IIFlow Cytometer and FACSAria cell sorter (BD Biosciences) for cell sorting.

Software

Data was analyzed using the Kaluza software (kaluza A85810 AB) (Beckman Coulter).

Cell population abundance

Cell populations were analyzed by gating on live B-ALL cells and single cell fractions depending on the experimental question.

Gating strategy

Cell populations were analyzed by gating on live B-ALL cells (FS/SS) and single cell fractions (FSC-A/FSC-H). Depending on the experimental question, different gating strategies were used and displayed and/or indicated in the respective figure (e.g. Figure 8b, Supplementary Figure 2c.).

- [x] Tick this box to confirm that a figure exemplifying the gating strategy is provided in the Supplementary Information.
